# Integrative Perspectives on Light-Regulated Metabolism in Medicinal Plants

**DOI:** 10.3390/metabo16070479

**Published:** 2026-07-08

**Authors:** Siqian Xiao, Dan Gao, Tielin Wang, Binbin Yan, Feng Xiong, Chongning Lv, Chuanzhi Kang

**Affiliations:** 1 State Key Laboratory for Quality Ensurance and Sustainable Use of Dao-Di Herbs, Institute of Chinese Materia Medica, China Academy of Chinese Medical Sciences, Beijing 100700, China; 2 College of Chinese Materia Medica, Shenyang Pharmaceutical University, Shenyang 110016, China; 3 National Resource Center for Chinese Materia Medica, China Academy of Chinese Medical Sciences, Beijing 100700, China

**Keywords:** medicinal plants, secondary metabolism, light signaling, light environment, metabolic regulation

## Abstract

**Background**: Light intensity and spectral quality are important abiotic factors that induce metabolic reprogramming in medicinal plants, thereby enabling adaptive responses to diverse radiation environments. This systematic review examines how different light conditions influence secondary metabolism, emphasizing that the observed differences can be interpreted as variations in response strategies and regulatory plasticity. **Methods**: Published transcriptomic and metabolomic studies on light-regulated secondary metabolism in medicinal plants were reviewed, with attention paid to light intensity, spectral quality, photoreceptor signaling, phytohormone interactions, and accumulation of major bioactive compounds. **Results**: Plants are often described as heliophytic or sciophytic; however, this classification primarily reflects ecological adaptations rather than inherent metabolic differences. Changes in metabolism are commonly associated with accumulation patterns of phenolic acids, terpenoids, alkaloids, and other bioactive compounds and are closely linked to dynamic regulation at the gene expression level. Based on transcriptomic and metabolomic evidence, we summarized signaling networks from photoreceptors, including phytochromes, cryptochromes, phototropins, and UV RESISTANCE LOCUS 8, to the Constitutive photomorphogenic 1–ELONGATED HYPOCOTYL 5 regulatory module and its interactions with JA, SA, and GA pathways. Variations in metabolic outcomes are mainly related to response thresholds, signaling intensity, and resource allocation, rather than exclusive production of specific metabolites. **Conclusions**: This review provides a framework for understanding light-regulated metabolic plasticity and offers insights into optimizing light environments and improving medicinal plant quality.

## 1. Introduction

Secondary metabolites (SMs) are not essential for basic cellular maintenance but play critical roles in mediating plant–environment interactions. These compounds contribute to plant defense, stress adaptation, and ecological fitness and are classified into three major groups: terpenoids, phenolics, and alkaloids [1]. Among these categories, terpenes and flavonoids represent important subclasses that are widely involved in plant defense and environmental adaptation. Among the environmental factors, light is one of the most influential regulators of SM biosynthesis. Beyond serving as the primary energy source for photosynthesis, light also acts as a key signaling cue that coordinates plant growth, development, and metabolic reprogramming. Accumulating evidence indicates that variations in light intensity and spectral composition can substantially alter the accumulation of SMs [2]. For example, enhanced flavonoid and phenolic accumulation have frequently been reported under high-light conditions, whereas reduced light often leads to adjustments in carbon allocation and shifts in metabolite profiles [3,4].

Transcriptomic and metabolomic approaches have opened up new avenues for elucidating the regulatory mechanisms underlying light-triggered secondary metabolism. Photoreceptors capture various light wavelengths, including phytochromes (PHYs), cryptochromes (CRYs), phototropins (Phots), and UV RESISTANCE LOCUS 8 (UVR8). These signals are further relayed through the central COP1–HY5 complex and PIF transcription factors, which interact with the JA, SA, and gibberellin (GA) hormonal pathways to jointly modulate the expression of metabolic genes [5,6,7,8,9,10]. Medicinal herbs display distinct responses to light irradiance; species such as *Astragalus membranaceus*, *Salvia miltiorrhiza*, and *Mentha haplocalyx* produce higher amounts of bioactive metabolites under high-light conditions, while *Panax ginseng* and *Fritillaria cirrhosa* perform better in shaded, low-light habitats [11,12,13]. Nevertheless, few integrated multi-omics investigations have fully established the causal links between upstream light signaling cascades and downstream metabolic reprogramming.

**Figure 1:**
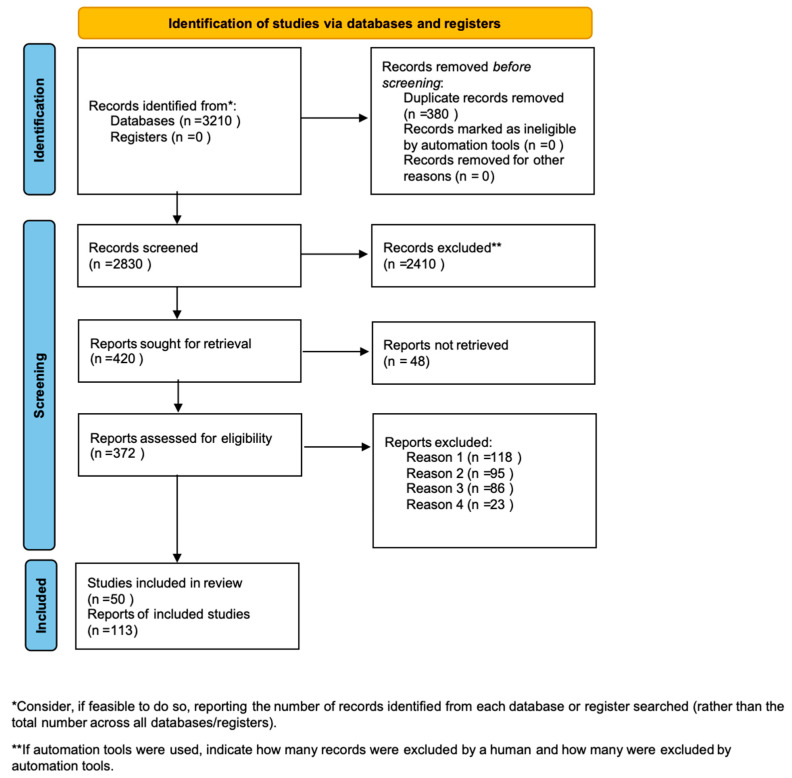
PRISMA 2020 flow diagram of literature screening for this new systematic review. Only electronic databases were searched; no clinical trial registers were retrieved, and the number of records from registers was recorded as zero. Additional information can be found in the Supplementary File S1 and S2.

**Figure 2:**
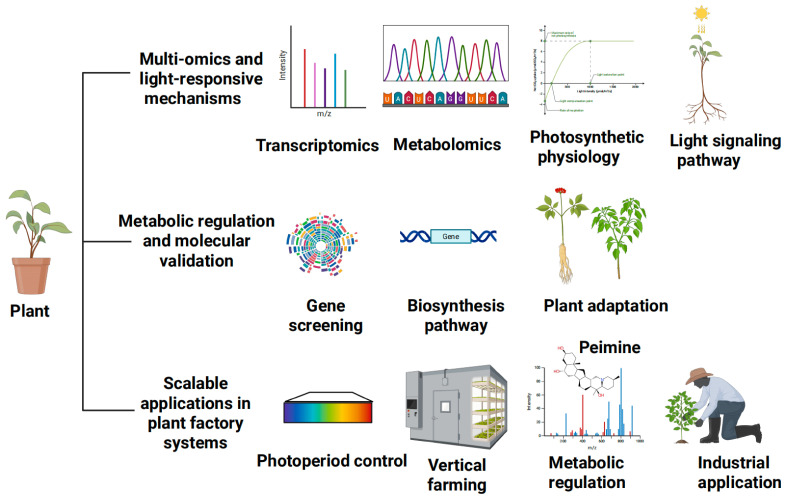
Multi-omics framework of light-regulated secondary metabolism in medicinal plants.

To ensure comprehensive coverage, literature was retrieved from databases, including Web of Science, Scopus, and PubMed, with a uniform retrieval time range spanning from database establishment to May 2026, using keywords such as “light signaling,” “secondary metabolism,” and “medicinal plants.” Clear literature inclusion and exclusion criteria were formulated for screening; only peer-reviewed English original articles, dissertations, and systematic reviews with complete physiological, multi-omics, or gene functional verification data related to the light-modulated secondary metabolism of medicinal plants were included. Conference abstracts, short communications, duplicate publications, and studies lacking valid light treatment experiments were excluded. All retrieved records underwent three-step standardized screening following the PRISMA guideline: duplicate removal via reference management software, preliminary screening by reading titles and abstracts, and full-text secondary screening. We further included classic landmark studies by backward-tracing the reference lists of highly cited reviews to avoid omissions. A PRISMA flow chart was attached to quantify the number of studies retained and eliminated at each screening stage [14]. Studies were selected based on their relevance to light-regulated metabolic processes, including experimentally validated studies and multi-omics-based analyses. Finally, we highlighted the potential applications of light-regulated cultivation strategies and synthetic biology approaches to improve the production and quality of medicinal plants (Figure 1 and Figure 2).

This figure illustrates the conceptual relationship among light perception, metabolic regulation, and practical applications in medicinal plants. Light signals are perceived by photoreceptors and analyzed using multi-omics approaches, including transcriptomics, metabolomics, and physiological measurements. These signals are integrated into the regulatory networks that control gene expression, biosynthetic pathways, and plant adaptive responses. The resulting metabolic outputs can be further modulated through optimized light conditions, enabling applications such as controlled cultivation systems and targeted enhancement of bioactive compounds.

## 2. Molecular Basis of Light Perception and Signal Transduction

### 2.1. Plant Photoreceptor Families (PHYs, CRYs, Phots, UVR8): Structure and Function

Plants have evolved a highly integrated photoreceptor system [14] that enables the perception of diverse light qualities, including red and far-red, blue, and UV-B wavelengths. This system mainly consists of PHYs, CRYs, Phots, and the UVR8, which collectively regulate development and metabolism. PHYs perceive red and far-red light through reversible photoconversion between their Pr and Pfr forms. This process was driven by conformational changes in the bilin chromophore. The Pfr form is biologically active and accumulates under red light conditions, while far-red light or darkness shifts the equilibrium toward the inactive Pr form. This reversible switching allows PHYs to function as dynamic molecular sensors of light environment [14,15] The photoactivated Pfr form of the PHYs translocates to the nucleus and interacts with PIFs. PIFs are key transcriptional regulators that integrate light signals with hormonal pathways, including auxin (IAA), GA, and brassinosteroid (BR) signaling. Depending on light conditions, PIF stability changes, leading to large-scale transcriptional reprogramming associated with growth regulation and metabolic adjustment [6].

CRYs (CRY1 and CRY2) are flavoproteins that perceive blue light through flavin adenine dinucleotide (FAD). Blue light induces conformational changes that inhibit the activity of E3 ubiquitin ligase COP1. This results in the stabilization of HY5, a central transcription factor involved in light-regulated development and secondary metabolism.

Phots contain light, oxygen, and voltage sensing (LOV) domains that bind to flavin mononucleotide (FMN). Blue-light activation triggers kinase activity, which regulates chloroplast relocation, stomatal opening, and phototropic growth. These processes contribute to the optimization of photosynthetic efficiency and carbon assimilation, indirectly affecting metabolic flux [16].

UVR8 is a UV-B-specific photoreceptor that directly absorbs UV-B photons via intrinsic tryptophan residues [17]. UV-B exposure induces monomerization of UVR8, which interacts with COP1 and promotes HY5-dependent transcriptional activation of UV-protective genes, particularly those involved in phenylpropanoid metabolism [18,19].

### 2.2. Core Hub of Light Signal Transduction: The COP1–HY5 Regulatory Module

Within the plant light signaling network, COP1 and the basic leucine zipper (bZIP) transcription factor HY5 form a central regulatory module that integrates multiple photoreceptor-derived signals and coordinates downstream developmental and metabolic responses. This module functions as a key signaling hub that links environmental light cues to transcriptional reprogramming.

In darkness or under extremely low-light conditions, COP1 predominantly localizes to the nucleus, where it assembles with SUPPRESSOR OF PHYA-105 (SPA) proteins to form an active E3 ubiquitin ligase complex. This complex selectively targets positive regulators of photomorphogenesis, including HY5, for ubiquitination and subsequent degradation via the 26S proteasome pathway. This represses light-responsive gene expression and minimizes energy expenditure in the absence of light [18]. Upon light exposure, activated photoreceptors, including PHYs and CRYs, interfere with COP1 activity, either by inducing conformational changes or by promoting its relocalization from the nucleus to the cytoplasm. These processes effectively reduce the COP1-mediated repression of HY5 [20]. Consequently, HY5 accumulates in the nucleus and functions as a pivotal transcriptional regulator.

HY5 directly binds to light-responsive *cis*-regulatory elements, such as G-box (CACGTG) and Activator of Cell Elongation (ACE) motifs, in the promoters of genes involved in secondary metabolism, including those responsible for the biosynthesis of anthocyanins, flavonoids, and terpenoids (e.g., chalcone synthase [CHS], chalcone isomerase [CHI], and flavonol synthase [FLS]) [21]. In medicinal plants, the functional status of the COP1–HY5 module largely determines the responsiveness of metabolic phenotypes to changes in the light environment. The abundance of HY5 protein generally shows a positive correlation with light intensity and may function as a quantitative “molecular rheostat” that modulates light-signal input. Under high-light conditions, elevated HY5 accumulation is often associated with the enhanced biosynthesis of antioxidant phenolic compounds, which can contribute to the alleviation of photooxidative stress [22]. Under shaded or low-light environments, changes in HY5 abundance and activity may alter its interactions with transcription factors, such as MYB and bHLH, thereby influencing metabolic flux distribution and the accumulation of specific SMs. These regulatory effects appear to be highly dependent on species, metabolite type, and environmental context [23]. Besides early core synthases that construct skeletons of phenolics and terpenoids, cytochrome P450 [CYP450] oxidoreductases act as core downstream catalysts for oxidative modification of herbal bioactive metabolites. Light-regulated CYP450 subtypes directly control the biosynthesis yield of tanshinones, artemisinin, paclitaxel and berberine, which are representative active ingredients in medicinal plants [24].

**Figure 3:**
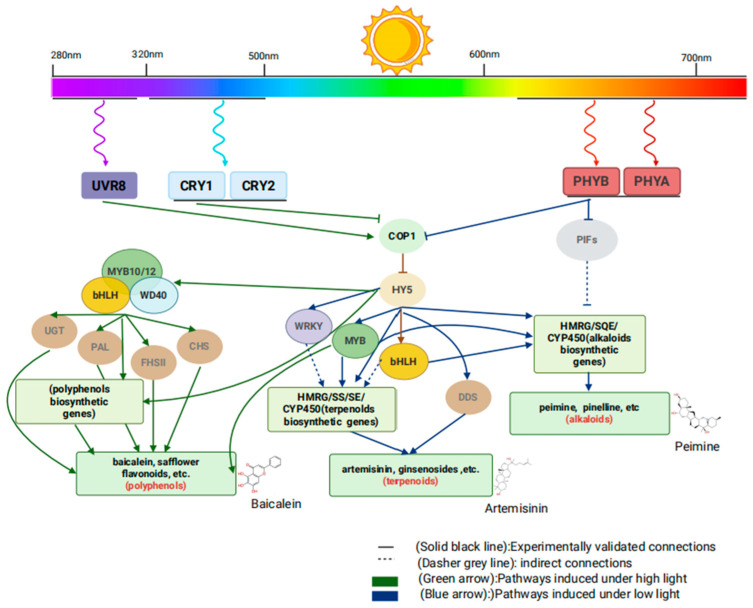
The COP1–HY5 hub integrates light signals to regulate secondary metabolism in medicinal plants. A schematic model of light signal regulation of SM biosynthesis in medicinal plants. Different light wavelengths are perceived by photoreceptors (UVR8, CRY1/2, PHYA/B), which regulate the core COP1–HY5 signaling module to control downstream transcription factors (MYB, bHLH, and WRKY). These factors further modulate three major secondary metabolic pathways (polyphenols, terpenoids, and alkaloids) via key biosynthetic genes/enzymes. Representative structures of pathway end products (baicalein, artemisinin, and peimine) are shown to indicate functional outputs. Abbreviations: COP1, Constitutive photomorphogenic 1; CRY, cryptochrome; HY5, ELONGATED HYPOCOTYL 5; PAL, phenylalanine ammonia-lyase; CHS, chalcone synthase; UGT, UDP-glycosyltransferase; HMRG, 3-hydroxy-3-methylglutaryl-CoA reductase gene; CYP450, cytochrome P450; PHY, phytochrome; UVR8, UV RESISTANCE LOCUS 8.

The COP1–HY5 module operates not merely as a binary molecular switch but also as a dynamic regulatory node that integrates environmental light signals into context-dependent metabolic outputs. This functional plasticity provides a mechanistic basis for the diversification of SM profiles in medicinal plants occupying different light ecological niches (Figure 3).

### 2.3. Crosstalk Between Light and Hormonal Signals (JA, SA, and GA) in Metabolic Regulation

Light signaling coordinates with phytohormone networks to regulate the balance among plant growth, defense, and secondary metabolism, constituting the central mechanism underlying the growth–defense trade-off [25]. Rather than acting independently of ecological classification, these responses are primarily driven by quantitative light cues such as irradiance, spectral composition, and shade signals, which are decoded by photoreceptors and integrated into hormone-regulated transcriptional networks [25].

JA is a central defense pathway that links light perception to SM biosynthesis. Under high-light or UV-B conditions, photoreceptor-mediated signaling enhances the expression of JA biosynthetic genes (e.g., lipoxygenase [LOX], allene oxide synthase [AOS]), leading to increased JA accumulation [22]. Degradation of ZIM-domain protein (JAZ) repressors enables activation of the transcription factor, myelocytomatosis protein 2 (MYC2), which drives the expression of JA-responsive genes involved in specialized metabolite production, including flavonoids, terpenoids, and alkaloids [23]. MYC2 cooperates with the light-responsive regulator HY5, thereby providing a direct transcriptional interface with light signaling and JA-dependent metabolic reprogramming [26]. In contrast, shade conditions mediated by reduced phytochrome B (phyB) activity promote the stabilization of PIFs, which enhances IAA and GA biosynthesis while maintaining JAZ-mediated repression of JA signaling, thereby shifting metabolism away from defense-oriented pathways toward growth promotion. Experimental evidence supports a strong coupling among JA signaling, light perception, and specialized metabolite production. In *Artemisia annua*, UV-B and JA signaling synergistically enhanced artemisinin biosynthesis through the coordinated activation of amorpha-4,11-diene synthase (ADS) and CYP71AV1 [27]. JA-responsive transcription factors, such as AaERF1 and AaERF2, directly regulate these biosynthetic genes. In addition, JA–MYC2 signaling promotes flavonoid and anthocyanin accumulation via MYB–bHLH transcriptional complexes across multiple plant species [23].

GA signaling further reinforces this trade-off through DELLA proteins, which function as central growth repressors. GA-induced DELLA degradation promotes cell elongation and growth [28], whereas DELLA accumulation under high-light conditions contributes to growth restraint and indirectly favors carbon allocation toward secondary metabolism. DELLA proteins also interact with JAZ repressors, creating a regulatory node that integrates the GA and JA pathways to fine-tune defense-related metabolic outputs [29,30]. The light quality further modulates this hormonal network via phyB–PIF signaling. Under shaded conditions, reduced phyB activity stabilizes PIF transcription factors, enhances IAA and GA biosynthesis, and maintains JAZ-mediated repression of JA signaling [31,32]. This shift promotes elongation growth while suppressing defense-related secondary metabolism, including flavonoid and phenolic compound accumulation.

SA signaling is also modulated by light via the redox-dependent regulation of its biosynthesis. Light-driven changes in photosynthetic electron transport influence the expression of isochorismate synthase 1 (ICS1), a key enzyme in SA biosynthesis [33]. Elevated SA levels under sufficient light enhance pathogen resistance through Non-expressor of Pathogenesis-Related Gene 1 (NPR1)-dependent signaling and promote phenylpropanoid metabolism, thereby increasing the accumulation of phenolic compounds via the activation of downstream biosynthetic genes [33].

Collectively, the JA-, GA-, and SA-centered signaling modules form interconnected regulatory hubs through which light cues are translated into transcriptional and metabolic outputs. These hubs converge on key transcriptional regulators, such as MYC2, PIFs, HY5, and NPR1, enabling the coordinated regulation of growth processes and secondary metabolic pathways in response to dynamic light environments. This integrated network supports flexible metabolic reprogramming, rather than fixed ecology-dependent regulatory modes.

## 3. Circadian and Photoperiodic Regulation of SM Dynamics and Harvest Timing

In the standardized cultivation of medicinal plants, harvest timing is conventionally determined solely based on the developmental stage or growing season. Accumulating metabolomic evidence demonstrates that the biosynthesis and accumulation of SMs are not static across a 24-h period; instead, they exhibit pronounced diurnal oscillations. These rhythmic patterns are co-regulated by the endogenous circadian clock and external environmental cues, particularly light and temperature, such that the metabolic profile of a single plant can differ substantially at pre-dawn, midday, and dusk [33]. Based on this, the concept of chronoharvesting has emerged as a chronobiology-guided strategy, in which plant materials are harvested at the daily peak of bioactive compound accumulation to maximize the content of pharmacologically active constituents in medicinal products.

Terpenoid constituents in essential oil-bearing medicinal plants display the most striking diurnal variation, with their dynamic abundance tightly linked to the diurnal supply of photosynthates and temperature-driven volatile loss [34]. For example, the essential oil content of *Mentha* species exhibits marked diurnal variation, with peak concentrations observed in morning-harvested samples. This pattern directly demonstrates the regulatory influence of diurnal rhythms on SM deposition [34]. In contrast, diurnal fluctuations in non-volatile phenolics and amino acid derivatives largely mirror the carbon-nitrogen allocation trade-offs modulated by light-dark cycles. As a representative woody medicinal species, *Camellia sinensis* possesses a sophisticated endogenous circadian network that integrates light signals to modulate the rhythmic transcription of genes underlying secondary phenolic metabolism, resulting in well-characterized diurnal metabolic profiles [35]. *C. sinensis* integrates circadian clock components to optimize growth, stress responses, defense mechanisms, and metabolic functions according to daily environmental fluctuations [36,37] to provide a systematic examination of the regulatory effects of the light environment on plant physiological metabolism.

Core circadian transcription factors intersect with light signaling cascades to orchestrate the diurnal expression profiles of genes involved in secondary metabolism. The core molecular mechanisms supporting this interplay were systematically described in the preceding section on light signal transduction and are not elaborated further here. This regulatory framework provides a theoretical foundation for the optimization of artificial photoperiod regimes and the implementation of precision harvesting practices in medicinal plant cultivation.

## 4. Metabolic Reprogramming Under High-Light Irradiance in Medicinal Plants

### 4.1. Oxidative Stress Induced by High-Light and Biosynthesis of Antioxidant SMs

High-irradiance conditions often exceed the capacity of the photosynthetic apparatus to utilize absorbed light energy, particularly when combined with environmental stresses such as drought and elevated temperatures. Under these conditions, the photosynthetic electron transport chain is over-reduced, increasing the probability of electron leakage and energy dissipation reactions [38]. Under such conditions, excited chlorophyll molecules cannot effectively dissipate energy through photochemical quenching. The excess energy can subsequently interact with molecular oxygen via electron leakage or energy transfer reactions, leading to the production of large amounts of reactive oxygen species (ROS), including singlet oxygen, superoxide anion, and hydrogen peroxide [39].

Although basal levels of ROS serve as pivotal signaling mediators in plant cells, surplus ROS generated under high-light stress, if left unneutralized, can induce lipid peroxidation of biomembranes, oxidative modification of proteins, and fragmentation of DNA strands. These oxidative lesions ultimately culminate in severe photoinhibition or photooxidative damage [40]. To mitigate such damage, plants activate enzymatic antioxidant systems, including superoxide dismutase (SOD), peroxidase (POD), and catalase (CAT). Plants also strongly trigger the production of antioxidant SMs, including flavonoids, anthocyanins, and diverse polyphenolics. These metabolites directly neutralize ROS and fulfill critical functions in plant photoprotection [41,42].

ROS can also act as core stress-signaling messengers. Through chloroplast-to-nucleus retrograde signaling cascades, ROS modulate nuclear gene expression and activate key genes associated with phenylpropanoid metabolism and flavonoid biosynthesis [39,43]. Under high-light stress, phenylalanine ammonia-lyase (PAL), the rate-limiting enzyme linking primary and secondary metabolic pathways, is first activated. Subsequently, the transcript levels of multiple core enzymes in the flavonoid biosynthetic pathway, including CHS, CHI, and FLS, were markedly elevated [44]. Consequently, a wide range of flavonoid compounds with strong antioxidant capacities accumulate in plant tissues.

Among these compounds, flavonols containing an ortho-dihydroxy structure [45], such as quercetin and luteolin and their glycoside derivatives, are particularly effective antioxidants. The hydroxyl moieties on the B-ring of these molecules can readily donate electrons or hydrogen atoms, thereby enabling them to directly reduce ROS and halt oxidative chain reactions. Consequently, flavonoids are recognized as one of the most crucial non-enzymatic antioxidant components in plants.

Furthermore, the accumulation of high-light-induced SMs often exhibits strong spatial specificity. These compounds are frequently preferentially localized in the vacuoles or cuticular layers of leaf epidermal cells [3]. This spatial distribution enables dual protective functions, namely intracellular ROS detoxification to maintain redox homeostasis and optical shielding through the absorption of UV-B and high-energy blue light, thereby reducing radiation reaching the mesophyll tissues and protecting photosystem II (PSII). High-light-induced ROS production triggers a conserved physiological response in plants that integrates antioxidant defense and secondary metabolic reprogramming. This response enhances plant tolerance to photooxidative stress and contributes to the accumulation of bioactive compounds in medicinal plants under strong light environments.

### 4.2. Photoprotection Mechanisms: Redirection of Secondary Metabolic Fluxes in Non-Enzymatic Antioxidant Systems

Under high-irradiance conditions, plants rely not only on low-molecular-weight antioxidant systems, such as ascorbate and glutathione, but also on the extensive reprogramming of primary metabolism to support the secondary metabolic pathways involved in photoprotection. When photosynthetic carbon fixation exceeds the growth demand, or when excess light energy disrupts the cellular NADPH/ATP balance, cells experience reductive pressure and redox imbalance [46]. Under such conditions, the photosynthetic electron transport chain remains in a highly reduced state, which favors the excessive production of ROS and consequently threatens cellular redox homeostasis. To restore metabolic balance, the carbon flux from primary metabolism is redirected toward secondary metabolic pathways, particularly the shikimate and phenylpropanoid pathways. In this strategy, carbon skeletons derived from primary metabolites, such as phosphoenolpyruvate and erythrose-4-phosphate, are extensively redirected through the shikimate pathway into phenylpropanoid metabolism and downstream specialized metabolic branches [44]. This metabolic reallocation effectively functions as a cellular “energy overflow valve.” Since the biosynthesis of SMs, including flavonols, anthocyanins, and phenolic acids, requires substantial energy and reducing power, enhanced flux through these pathways continuously consumes excess NADPH and ATP. In *S. miltiorrhiza* subjected to gradient light intensity treatments, KEGG pathway enrichment analysis demonstrates that primary metabolic pathways, including glycolysis and the pentose phosphate pathway, are transcriptionally downregulated with rising light intensity, while phenylpropanoid and flavonoid biosynthetic pathways are significantly upregulated. Integrated gene-metabolite co-expression network analysis, combined with published functional validation evidence, identifies SmMYB111 as a core transcription factor supporting phenolic acid biosynthesis under high-light conditions. The transcript abundance of SmMYB111 shows a significant positive correlation with rosmarinic acid and salvianolic acid B accumulation. This regulator mediates the transduction of light-intensity signals into secondary metabolic reprogramming, establishing a complete response cascade spanning light-intensity input, transcriptional regulation, and final metabolite accumulation. Such a global regulatory network cannot be fully resolved using single-omics approaches alone [44]. In *Scutellaria baicalensis*, flavonoids, including baicalein and wogonin, exhibit strong responsiveness to elevated irradiance [47]. In *Lonicera japonica*, chlorogenic acid and luteolin derivatives increase with increasing light intensity [48,49]. In *Carthamus tinctorius*, high-light levels enhance the biosynthesis and accumulation of chalcone pigments, such as hydroxysafflor yellow A [44]. In addition to metabolic flux redirection, enhanced SM accumulation contributes to photoprotection by reducing excessive excitation pressure on the photosynthetic apparatus. For example, in *Ginkgo biloba*, high-light and UV exposure upregulate GbCHS and GbFLS expression, thereby promoting flavonol glycoside biosynthesis [50]. The resulting accumulation of quercetin and kaempferol derivatives, primarily localized in epidermal cells, forms a physical “light-shielding layer” and acts as an efficient ROS scavenging system that protects PSII from excessive light-induced damage.

### 4.3. Light-Intensity Dose–Response Relationships in Medicinal Plants

In medicinal plants, the relationship between light intensity and SM accumulation is nonlinear and typically follows a dose–response pattern characterized by an optimal operational range and a saturation threshold. Within the optimal range, increasing irradiance enhanced photosynthetic efficiency and carbon assimilation, thereby promoting the redistribution of carbon flux toward secondary metabolic pathways. When the light intensity exceeds the physiological threshold, photoinhibition and photooxidative stress occur, leading to impaired photosynthetic electron transport and restricted metabolic productivity. This dose-dependent behavior has been broadly observed across plant species and reflects a conserved regulatory principle in plant light adaptation, in which metabolic outputs are determined by the balance between energy input and cellular redox homeostasis rather than by species-specific ecological categories. At moderate irradiance, carbon skeletons from primary metabolism are efficiently redirected to phenylpropanoid, flavonoid, and terpenoid biosynthetic pathways, supporting the accumulation of diverse SMs. Under excessive light conditions, oxidative stress and reduced photosynthetic efficiency limit carbon fixation and shift metabolic priorities toward protective and repair processes. At the molecular level, this dose–response regulation is mediated by coordinated transcriptional networks that integrate light perception, hormone signaling, and metabolic regulation. Light-responsive transcription factors, such as HY5 and bZIP, link light intensity signals to downstream metabolic gene expression. In parallel, stress-responsive signaling pathways modulate the expression of key biosynthetic enzymes involved in secondary metabolism, including those associated with flavonoid and phenylpropanoid biosynthesis.

In *G. uralensis*, light quality and intensity regulate SM accumulation through the coordinated modulation of photosynthetic performance and biosynthetic gene expression. Red and red–blue combined light conditions enhance plant growth and promote the accumulation of glycyrrhizic acid-related metabolites via the upregulation of biosynthetic genes [50,51,52]. In contrast, blue light and UV-B exposure trigger metabolic reprogramming associated with stress responses, including activation of antioxidant pathways and SM biosynthesis [50,51].

The Asteraceae medicinal herb *C. tinctorius* exhibits light-responsive molecular mechanisms involving transcription factors and gene families that regulate growth, development, and SM biosynthesis. Liang and Wang identified 52 bZIP genes in safflower and demonstrated that the transcript levels of several bZIP transcription factors were modulated by light intensity, suggesting their roles in light signal transmission and hormone-dependent pathways [53]. HY5 has been shown to promote flavonoid accumulation by upregulating structural genes, such as CHS, under varying light intensities [54]. In addition, the light-responsive expression patterns of flavonoid biosynthetic enzymes and CYP450 family genes further indicate coordinated regulation between light perception and secondary metabolism [55,56]. Metabolomic analysis via GC-MS identified sesquiterpenoid volatile oil markers across five Atractylodes species, whose biosynthesis is tightly modulated by light intensity and acts as representative terpenoid SM examples to enrich the light dose-response dataset [50] (Table 1).

## 5. Metabolic and Regulatory Responses of Medicinal Plants to Low-Light Environments

### 5.1. Shade Avoidance Syndrome (SAS) and Trade-Offs in SM Allocation

Heliophytes exhibit physiological and developmental adjustments in low-light and canopy-shaded environments. When canopy shading decreases the red/far-red (R/FR) ratio below a critical threshold, plants may still activate SAS to enhance light capture. This response is often accompanied by resource allocation tradeoffs between growth and secondary metabolism. At the molecular level, low R/FR signals induce the conversion of the photoreceptor phyB into its inactive Pr form, thereby releasing the repression of PIFs, which subsequently accumulate in the nucleus [63]. Accumulated PIFs have been proposed to function as important regulators coordinating growth-related processes and secondary metabolic responses under low R/FR conditions [64]. On the one hand, PIFs can activate the expression of IAA biosynthetic genes (YUCs) and GA biosynthetic genes, promoting cell elongation and vegetative growth [10]. On the other hand, PIF signaling has been associated with the suppression of MYC2 activity and the stabilization of JAZ proteins, which may attenuate JA-mediated secondary metabolic defense pathways [65]. Collectively, these responses suggest that low-light signaling can influence the balance between plant growth and SM accumulation in a species- and context-dependent manner.

### 5.2. Regulation of Terpenoids, Saponins, and Alkaloids Biosynthesis Under Low-Light Environments

Under low-light conditions, sciophytes adjust photosynthetic efficiency and carbon-nitrogen distribution to maintain metabolic balance [39]. This environment exerts specific regulatory effects on terpenoids, saponins, and alkaloids. These three metabolite groups rely less on light-triggered antioxidant protection than on phenolic compounds. Strong light increases ROS production, which restricts key enzymes in the mevalonate (MVA) pathway at the post-transcriptional level [66]. Moderate–low light intensity weakens this oxidative inhibition and helps maintain the formation of lipophilic storage-accumulated SMs. In *P. ginseng* subjected to gradient shading regimes, integrated transcriptomic and metabolomic analyses revealed that as light intensity declined, pathways involved in photosynthetic carbon fixation maintained stable transcriptional activity, whereas the MVA pathway-mediated triterpenoid saponin biosynthetic pathway was progressively upregulated. Using weighted gene co-expression network analysis (WGCNA), the study further pinpoints PgWRKY38 as a hub transcription factor that supports saponin biosynthesis under low-light conditions. The transcript abundance of PgWRKY38 is strongly correlated with the accumulation levels of ginsenosides Rg1 and Rb1. This work systematically delineates the metabolic regulatory network underlying the response of shade-adapted medicinal plants to light intensity variations [59,67]. Red and blue light activate these biosynthetic genes, while far-red light affects saponin accumulation in a tissue-specific and development-dependent way. This shows that light quality modulates metabolic flow instead of forming fixed metabolite profiles [67]. Transcription factors, including MYB and WRKY (e.g., PnMYB31, PnWRKY38), interact with HY5 and jointly control saponin formation under low light [60,61,62]. Low-light exposure also affects steroidal and medicinal alkaloid levels. In *F. cirrhosa*, low-light signals pass through the COP1–HY5 pathway, activate MYB and WRKY transcription factors, and upregulate MVA pathway genes. This process promotes the accumulation of steroidal alkaloids such as fritillarine [68]. In *P. ternata*, proper shading coordinates the expression of photosynthetic and TCA cycle genes through HY5. This coordination provides a suitable metabolic condition for tuber development and alkaloid formation [62].

The regulatory patterns of low light on terpenoids, saponins, and alkaloids were consistent across species. Moderate–low light relieves the oxidative damage caused by strong light, optimizes carbon and energy distribution, and activates biosynthetic pathways via light signaling and transcription factor regulation [69,70]. This pattern is a common adaptive mechanism in shade-growing medicinal plants and is not unique to triterpenoid saponins.

### 5.3. Ecological and Spectral Effects of Diffuse and Canopy-Filtered Light on Understory Medicinal Plants

In forest ecosystems, understory light environments are characterized by reduced irradiance and altered spectral composition resulting from canopy filtering [71]. Collectively, these factors influence the physiological performance and quality of understory medicinal plants. Compared with direct light, diffuse light transmitted through or reflected by the canopy provides more uniform illumination to the middle and lower leaf layers, potentially reducing excessive self-shading and photodamage. *Paris polyphylla* exhibited optimal growth under approximately 50–70% shading, which minimizes damage to photosystem II while avoiding the adverse effects associated with either excessive irradiance or insufficient light [72].

Canopy filtering also modifies the spectral composition by preferentially absorbing red and blue wavelengths while allowing relatively higher proportions of green and far-red light to reach understory habitats. These spectral changes can influence the photosynthetic regulation and pigment composition. Enhanced utilization of green light has been associated with adjustments in chlorophyll composition, including altered chlorophyll a/b ratios, which may improve light-harvesting efficiency under shaded conditions [73].

Rather than acting as fixed determinants of plant metabolic types, diffuse and canopy-filtered light function as environmental regulators that influence photosynthetic performance, metabolic allocation, and SM accumulation. These responses arise from interactions among light intensity, spectral quality, plant developmental status, and physiological plasticity (Table 2).

## 6. Light Quality-Specific Regulation of SMs

### 6.1. UV-B Radiation and Stress-Induced Accumulation of Phenolics and Alkaloids

Ultraviolet radiation, particularly UV-B (280–315 nm), has long been regarded as a stress factor that causes DNA damage and disrupts the photosynthetic apparatus in plants. However, recent studies on plant physiology have demonstrated that low doses of UV-B radiation can act as one of the most effective abiotic elicitors for inducing the biosynthesis of SMs in medicinal plants [17]. This effect can be explained by the concept of light–stress interactive adaptation. Plants perceive UV-B signals through the specific photoreceptor UVR8, which subsequently activates the downstream transcription factor HY5 and triggers transcriptional reprogramming of the phenylpropanoid pathway [78]. Consequently, plants enhance the biosynthesis of phenolic acids and flavonoids with strong UV-absorbing properties, thereby forming a molecular “UV screen” in epidermal cells that helps protect tissues from excessive ultraviolet radiation [79].

In *S. miltiorrhiza*, UV-B exposure enhances phenolic acid biosynthesis by upregulating key enzymes in the phenylpropanoid pathway [81]. Yin et al. further demonstrated that UV-B treatment significantly increased the transcription of genes involved in rosmarinic acid biosynthesis [80]. UV-B radiation regulates phenolic acid biosynthesis through transcriptional and post-translational mechanisms, including the stabilization of rosmarinic acid synthase and the suppression of negative regulatory factors involved in phenolic metabolism [81]. UV-B exposure enhances antioxidant enzyme activity and increases phenolic compound accumulation, thereby alleviating oxidative damage and improving stress tolerance in plants [82].

Beyond phenolic metabolism, UV-B signaling affects alkaloid biosynthesis, as demonstrated by enhanced terpenoid indole alkaloid accumulation following UV-B treatment in *C. roseus* [83]. This effect is generally considered to involve broader metabolic reprogramming, including changes in energy metabolism and stress-responsive signaling networks, rather than pathway-specific induction alone [83,84].

### 6.2. Blue Light-Mediated Transcriptional Activation of Key Enzyme Genes in Terpenoid Biosynthesis

Within the secondary metabolic regulatory network of medicinal plants, blue light (400–500 nm) is widely acknowledged as one of the most potent spectral cues driving terpenoid biosynthesis. This regulatory process mainly relies on signal transduction mediated by the blue-light photoreceptors. Upon blue-light activation, CRYs inhibit the activity of COP1, thereby blocking the proteasomal degradation of the transcription factor HY5. Nuclear accumulation of HY5 then triggers the transcription of downstream genes involved in terpenoid biosynthesis [85]. This regulatory effect is largely associated with the CRY-mediated suppression of COP1 and the stabilization of HY5, which subsequently activates the transcription of genes involved in the MVA and MEP pathways [85]. For instance, HMGR, a core rate-limiting enzyme in the MVA pathway, harbors blue-light-responsive elements in its promoter, which endow it with high sensitivity to blue-light cues, facilitating the activation of the terpenoid biosynthetic pathway.

Zhang et al. reported that blue light stimulated the transcription of core genes in the artemisinin biosynthetic pathway of *A. annua*, including ADS and cytochrome P450 716AV1 (CYP716AV1), thus boosting artemisinin accumulation without compromising plant growth [86,87]. Furthermore, overexpression of the blue-light receptor *A. annua* cryptochrome 1 (AaCRY1) leads to up to a 2.4-fold increase in artemisinin content, highlighting the pivotal molecular role of blue-light signaling in the regulation of secondary metabolism [88]. In *Mentha* species subjected to blue-light irradiation, integrated transcriptomic and metabolomic analyses corroborated the inductive effect of blue-light on monoterpene biosynthesis and identified MhMYC2 as a critical regulatory transcription factor via the construction of a gene-metabolite co-expression network. Further molecular functional validation confirmed that MhMYC2 forms a protein complex with MhHY5, a core light-signaling regulator, to coordinately modulate the biosynthesis of key essential oil constituents, including limonene and menthol. Additionally, the study revealed that blue light concurrently regulated the expression of genes associated with glandular trichome development. It systematically delineated the full mechanism of blue-light-regulated essential oil accumulation at three hierarchical levels, namely organ development, biosynthetic pathways, and metabolite accumulation, overcoming the inherent limitations of single-omics approaches that can only resolve alterations in individual pathways [36]. On the basis of 70% red + 30% blue light, supplemental far-red (FR) greatly boosted mint biomass and PSII photosynthetic performance, whereas UVA showed little influence. FR induced massive transcriptional and metabolic reprogramming, enriching phenylpropanoid and flavonoid pathways to raise aromatic volatiles (methylchavicol, anethole) and antioxidant flavonoids [13].

### 6.3. Fine-Tuned Regulation of Metabolic Pathways by Red/Far-Red Ratios via Photoreceptors

The R/FR light ratio is a key environmental signal that reflects the canopy structure and plant competition. Changes in R/FR ratio have been widely reported to influence both growth and secondary metabolism in medicinal plants [19,89]. PhyB functions as the primary sensor of R/FR signals, regulating the stability of PIF transcription factors and influencing transcriptional networks associated with carbon allocation and metabolic activity [89].

In *A. thaliana*, altered R/FR ratios have been shown to affect both growth-related traits and SM accumulation, particularly under shade conditions [90,91,92]. Similar regulatory patterns have been reported in medicinal plants, although responses varied significantly across species. In *P. ginseng*, light spectral composition has been shown to influence ginsenoside accumulation, with red-enriched light often associated with increased triterpenoid saponin biosynthesis, likely through effects on photosynthetic performance and subsequent metabolic flux [67,93]. In *P. ternata,* alterations in light quality have been documented to affect alkaloid production and tuber formation, suggesting that light signaling may synchronize growth and metabolic processes [94] (Table 3).

## 7. Conclusions and Future Perspectives

### 7.1. From Single Signals to Multidimensional Networks: Research Limitations and Insights into Epigenetic and Post-Transcriptional Regulation

Current studies on light-regulated secondary metabolism in medicinal plants have gradually established a regulatory framework centered on the “photoreceptor–transcription factor–structural gene” cascade. Most recent investigations have concentrated on the static stimulatory effects of individual light qualities or fixed light intensities on particular metabolites. Systematic investigations into the real-time responses of metabolic fluxes in dynamic light environments, such as transient fluctuations of light intensity and spectral composition commonly occurring in nature, remain limited. Moreover, most multi-omics studies rely on simple linear correlations between transcriptomic and metabolomic datasets, which are insufficient to reveal the complex nonlinear regulatory mechanisms underlying light-signal transduction networks. In particular, the modulatory functions of post-translational protein modifications and metabolite feedback control remain unclear.

Another emerging, but still debated, concept is the so-called “metabolic memory.” This concept was originally proposed for human disease research, particularly in diabetes, where transient metabolic or environmental stimuli can induce long-lasting epigenetic alterations and persistent transcriptional changes [95]. Inspired by this framework, similar hypotheses have been proposed in plants, suggesting that transient light exposure may leave molecular imprints at the chromatin level, potentially involving changes in DNA methylation and histone modifications, such as histone H3 lysine 4 trimethylation (H3K4me3) and histone H3 lysine 27 trimethylation (H3K27me3), in genes related to secondary metabolism [96,97]. In addition, post-transcriptional regulation, particularly light-regulated alternative splicing, may generate transcription factors or enzyme isoforms with distinct functions [11,98]. Such mechanisms may play important yet underestimated roles in the adaptation of shade-tolerant plants to heterogeneous understory light conditions and in the fine regulation of metabolic trade-offs. Future research should, therefore, move beyond single-signal analyses toward integrated multidimensional regulatory networks, incorporating epigenetic and post-transcriptional layers, to better understand how light signals shape the metabolic phenotypes of medicinal plants.

### 7.2. Precision Cultivation in Plant Factories Based on “Light Recipes”

With the rapid development of controlled environmental agriculture (CEA), translating the fundamental knowledge of plant photobiology into practical applications has become increasingly important. A key strategy is to establish species- or chemotype-specific “dynamic light recipes” [35]. Future plant factory systems are expected to move beyond the static optimization of red–blue light ratios and instead adopt dynamic lighting strategies that better simulate natural light fluctuations. These systems can regulate the spectral composition, photosynthetic photon flux density (PPFD), and photoperiod according to developmental stages and circadian rhythms [98]. For instance, the application of short-term high-intensity UV-B or blue-light “stress pulses” prior to harvest can significantly increase the accumulation of target bioactive compounds without markedly reducing biomass [98]. Integrating large datasets linking genotype–light environment–metabolic phenotypes with artificial intelligence prediction models may enable a transition from empirical cultivation to digitally controlled precision production, thereby addressing challenges such as unstable quality, heavy metal contamination, and pesticide residues in medicinal materials, while facilitating the industrial production of high-quality medicinal plants [100]. GWAS strategies can decode the genetic basis of high secondary metabolite traits in ideal medicinal herbs, providing genotypic reference for designing species-specific dynamic light recipes in plant factory precision cultivation [110].

### 7.3. Potential of Synthetic Biology and Gene Editing to Improve Light Use Efficiency and Metabolic Quality

Traditional breeding strategies have long been constrained by the intrinsic trade-off between plant growth and secondary metabolism, which is particularly evident under suboptimal light conditions [101]. The rapid development of synthetic biology and CRISPR/Cas-based genome editing technologies has opened up new opportunities to reprogram plant light-response pathways, with the aim of simultaneously enhancing photosynthetic efficiency and SM production.

A key challenge is that the central components of light signaling networks, such as photoreceptors (e.g., PhyB and cryptochromes) and key transcription factors (e.g., HY5 and MYC2), often function as regulatory hubs [72]. Previous studies have shown that the direct manipulation of these core regulators frequently induces extensive pleiotropic effects and unintended transcriptional reprogramming, potentially compromising plant developmental stability and environmental adaptability. Consequently, overcoming the growth–defense trade-off through genetic engineering relies not only on advanced technological tools but also on the rational design of more precise and controllable regulatory strategies.

To address these challenges, future studies should focus on precise regulation, context-dependent control, and modular engineering strategies. First, compared with complete gene knockout or constitutive overexpression, quantitative modulation of photoreceptor activity is a more feasible approach. Strategies such as *cis*-regulatory element editing, promoter engineering, and allele-specific modification can fine-tune light sensitivity or spectral responsiveness while minimizing the disruption to downstream signaling networks [104]. Such expression-level regulation alleviates pleiotropic effects associated with key regulatory factors, thereby enabling relatively high metabolic activity under low-light conditions or artificial lighting systems. Second, for downstream transcription factors, such as HY5 and MYC2, spatial- and temporal-specific regulation is considered an effective strategy to reduce the pleiotropic effects [99]. The use of tissue-specific promoters, inducible expression systems, and environment-responsive regulatory elements allows for fine control over the balance between growth and secondary metabolism in specific tissues or under defined conditions, thereby decoupling these two processes without globally perturbing plant physiology [105]. Finally, synthetic biology offers an alternative and promising strategy for constructing orthogonal light-responsive metabolic modules [105]. These artificially designed genetic circuits can operate partially independently of endogenous regulatory networks, thereby reducing the reliance on the complexity of native signaling pathways. For instance, the introduction of light-inducible synthetic gene clusters into suitable plant chassis systems or heterologous hosts may enable more precise and controllable production of target bioactive compounds. As a representative heterologous biosynthesis case for TCM compound preparations, engineered Saccharomyces cerevisiae (Compound Danshen Yeast 1.0) can co-produce multiple signature bioactive compounds of Compound Danshen, providing a sustainable strategy to reduce wild medicinal plant exploitation [111].

Advances in high-fidelity Cas variants and base-editing technologies have improved the precision of genome editing [11], reducing off-target effects and enhancing the safety and reliability of genetic modifications. Overall, future directions in this field are expected to shift away from the broad reprogramming of central regulatory hubs toward more refined, modular, and orthogonal engineering strategies. Within this framework, it may become possible to simultaneously improve light-use efficiency and SM production in medicinal plants, while minimizing pleiotropic and unintended developmental effects.

### 7.4. Endemicity Predictions Under Climate Change and Conservation of Rare Understory Sciophytes

**Figure 4:**
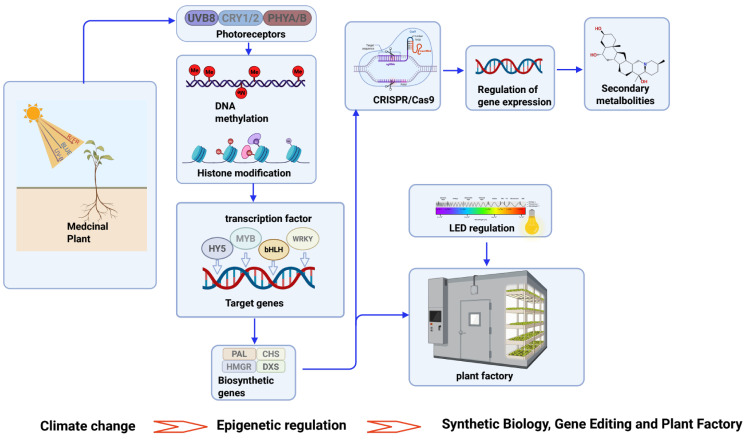
Integrative regulation of secondary metabolism by light: epigenetic, transcriptional, and biotechnological perspectives. This diagram illustrates the complete logic of light regulating secondary metabolism in medicinal plants through three pathways: epigenetic regulation, transcriptional control, and biotechnological applications. Light signals, perceived by photoreceptors, regulate transcription factors via epigenetic modifications to activate secondary metabolic biosynthetic genes. Further, gene editing or LED light regulation in plant factories enables precise control and industrial application of secondary metabolism.

Climate change is gradually altering forest environments by reshaping canopy structures, increasing habitat fragmentation, and altering atmospheric conditions. These shifts affect the amount of light reaching the understory, in terms of intensity and spectral composition [112]. Processes such as forest disturbances that create canopy gaps, together with changes in ultraviolet-B radiation linked to ozone variation, contribute to this variability [109]. Shade-adapted medicinal plants, such as *P. ginseng*, can respond sensitively to such changes, showing alterations in growth and SM profiles when light conditions become less stable. The fourth national survey of Chinese materia medica resources systematically cataloged more than 15,000 medicinal plant species in China, covering a large number of rare shade-dependent medicinal taxa threatened by climate change, supplying authoritative basic data for resource sustainable utilization [113]. Similar responses have been observed in many non-medicinal understory species, suggesting that this is a general ecological response rather than a feature unique to medicinal plants. In this sense, climate change acts mainly as an external driver that modifies the light environment, which in turn influences plant physiological and metabolic regulation through established light-signaling pathways. The multi-layer regulatory network of light signaling in plants and its applications in plant factory cultivation and genetic engineering improvements are illustrated in Figure 4. This review systematically summarizes the core regulatory mechanisms that cover the complete pathway from plant light signal perception to the synthesis of specialized SMs, providing a theoretical reference for subsequent mechanistic research and precise utilization of medicinal plant resources.

## Figures and Tables

**Table 1:** Molecular mechanisms of medicinal plants under high-light.

Family	Species	SMs	Light Treatment	Molecular Mechanisms	Reference
Asteraceae	*Carthamus tinctorius*	Phenolics	250, 120 μmol·m^−2^·s^−1^ PPFD	Light activates COP1–HY5 via photoreceptors, regulating MYB and WRKY to promote key phenylpropanoid–flavonoid genes (PAL, C4H, 4CL, CHS, CHI, F3H, FLS) and HSYA accumulation.	[57]
Caprifoliaceae	*Lonicera japonica*	Flavonoids	100%, 50%, 25% full light	Light activates the COP1–HY5 signaling module via photoreceptors, regulating MYB, bHLH, and WRKY transcription factors to promote the expression of key phenylpropanoid and flavonoid pathway genes (PAL, C4H, 4CL, CHS), thereby enhancing the accumulation of chlorogenic acid and flavonoids in *L. japonica*.	[51,52,58]
Lamiaceae	*Perilla frutescens*	Anthocyanin pigments	120 μmol·m^−2^·s^−1^ PPFD	Upregulates the biosynthetic pathway of anthocyanin pigments in Perilla frutescens suspension cells;	[59]
Lamiaceae	*Rosmarinus officinalis*	Volatile oil	100% full light	100% full light upregulates pinene synthases and bornyl diphosphate synthases, two key enzyme families in volatile oil biosynthesis; full sunlight elevates the relative abundance of α-/β-pinene, camphene, myrcene, borneol, camphor and bornyl acetate	[60]
Ranunculaceae	*Coptis chinensis*	Benzylisoquinoline alkaloids	50 μmol·m^−2^·s^−1^ PPFD	Photoreceptor-mediated light signaling regulates WRKY, bHLH, and MYB transcription factors, which control key genes in the benzylisoquinoline alkaloid pathway (TYDC, NCS, BBE) and promote berberine biosynthesis	[61,62]

**Table 2:** Molecular mechanisms of medicinal plants under low light.

Family	Species	SMs	Light Treatment	Molecular Mechanisms	Reference
Araceae	*Pinellia ternata*	Succinic acid	55% full light (low light)	Low light promotes tuberization and alkaloid accumulation; coordination of photosynthesis and TCA cycle genes (RBCS1, GGAT2, CAB7, Lhca4, Lhcb5, PsaD, PsaF, and PsbY) is mediated by the transcription factor HY5.	[74]
Araliaceae	*Panax ginseng*	Ginsenosides	100 μmol·m^−2^·s^−1^	upregulated the expression of HMGR, SS, CYP716A53v2, UGT74AE, PgUGT1, and UGTPg45	[74]
Araliaceae	*Panax notoginseng*	Flavonoids	15% full light	upregulates the saponin biosynthesis pathway; involvement of the HY5–WRKY module in its regulation.	[47,75,76]
Araliaceae	*Panax quinquefolius*	Ginsenosides	80; 120 μmol·m^−2^·s^−1^	It mediates the biosynthesis and accumulation of ginsenosides in *Panax quinquefolius* by regulating the MVA/MEP pathways and key downstream genes involved in triterpenoid saponin biosynthesis.	[77]
Aristolochiaceae	*Asarum heterotropoides*	Volatile oil, Asarinin	50% full light	50% full light upregulates cinnamyl alcohol dehydrogenase and cytochrome p450719As to promote the biosynthesis of volatile oil and asarinin	[78,79]
Zingiberaceae	*Curcuma xanthorrhiza*	β-bisabolol, Curzerene, Curcuphenol, γ-himachalene	75% shade light	By activating MVA/MEP terpenoid backbone pathways and upregulating sesquiterpene-specific synthases, 75% shade light promotes exclusive biosynthesis of β-bisabolol, curzerene, curcuphenol and γ-himachalene volatile sesquiterpene secondary metabolites.	[80]

**Table 3:** Molecular mechanisms of medicinal plants under different light qualities.

Family	Species	SMs	Light Treatment	Molecular Mechanisms	Reference
Apocynaceae	*Catharanthus roseus*	Monoterpenoid indole alkaloids (vinblastine, vincristine)	Red2:Blue1; Red7:Blue1	Light activates COP1–HY5 via photoreceptors, regulating WRKY, ORCA, MYC2, and CrGRF1/4 to promote the expression of key alkaloid biosynthetic genes, enhancing the accumulation of vinblastine and vincristine.	[37,95,96,97]
Araliaceae	*Panax ginseng*	Total saponin; protopanaxatriol -type ginsenosides	Blue light, Red light(LED)	Blue light upregulated the expression of HMGR, SS, SE, DS, CYP716A52, and CYP716A47, and the expression of HMGR, SS, SE, DS, and CYP716A47 under red light treatment was significantly upregulated in *P. ginseng* roots.	[72]
Aristolochiaceae	*Asarum heterotropoides*	Phenylpropanoids; essential oil	Red; Yellow; Blue; green; Purple and 50%sunlight	By regulating the expression of key enzymes in the shikimate and phenylpropanoid pathways (PAL, C4H, and 4CL), the biosynthesis of volatile oils and lignan SMs is promoted.	[98]
Asteraceae	*Artemisia annua*	Artemisinin	Red light	Light signals activate COP1–HY5 via photoreceptors, regulating WRKY and ERF to promote the expression of key artemisinin biosynthetic genes and their accumulation.	[27,99,100,101]
Asteraceae	*Artemisia argyi*	Total phenols, flavonoids, jaceosidin,	Blue light	Blue light upregulates blue light photoreceptors to maintain high photosynthetic efficiency (ΦPSII, ETR(II), Fv/Fm) with low energy dissipation	[102]
Caprifoliaceae	*Lonicera japonica*	Chlorogenic acid, luteoloside	Blue light	Light activates the COP1–HY5 signaling module via photoreceptors, regulating MYB, bHLH, and WRKY transcription factors to promote the expression of key phenylpropanoid and flavonoid pathway genes (PAL, C4H, 4CL, CHS), thereby enhancing the accumulation of chlorogenic acid and flavonoids in *L. japonica*.	[103]
Caprifoliaceae	*Mentha haplocalyx*	Monoterpenoids (volatile oils, menthol, menthone	Blue:Red = 3:7 + FR	Light regulates glandular trichome development and transcription factors (e.g., MYB), thereby promoting the expression of key monoterpene biosynthetic genes and enhancing menthol and menthone accumulation in *Mentha haplocalyx*.	[13,92]
Fabaceae	*Astragalus membranaceus*	Triterpenoid saponins and isoflavonoids (astragalosides, calycosin)	White light	Light regulates MYB transcription factors (e.g., AmMYB12), promoting the expression of key triterpenoid saponin and isoflavonoid pathway genes (CHS) and thereby enhancing the accumulation of astragalosides and isoflavonoids in A. membranaceus.	[104,105]
*Lamiaceae*	*Salvia miltiorrhiza*	Diterpenoids and phenolic acids (tanshinones, salvianolic acids)	Blue:Red = 3:7	Light activates the HY5 regulatory network via photoreceptors, regulating MYB and bHLH transcription factors to promote key terpenoid and phenylpropanoid pathway genes, thereby enhancing tanshinone and phenolic acid accumulation in *S. miltiorrhiza*.	[11]
Lamiaceae	*Scutellaria baicalensis*	Flavonoids (baicalin, baicalein)	Blue light	Light regulates MYB transcription factors (e.g., SbMYB45 and SbMYB86), promoting the expression of key phenylpropanoid-flavonoid pathway genes (PAL, CHS, CHI, FNSII) and thereby enhancing the accumulation of baicalin and baicalein in *S. baicalensis*.	[106]
Lamiaceae	*Rosmarinus officinalis*	Volatile oil, rosmarin	Red and far-red	Red and far-red light differentially regulate terpene synthase genes to modulate volatile oil components	[107]
Liliaceae	*Fritillaria cirrhosa*	Steroidal alkaloid	Blue light	Light signals are perceived by photoreceptors and transmitted through the COP1–HY5 signaling module, which regulates transcription factors such as MYB and WRKY, thereby modulating the expression of key genes in the MVA pathway and ultimately promoting the biosynthesis and accumulation of steroidal alkaloids.	[68]
Orchidaceae	*Anoectochilus roxburghii*	Flavonoids	Red:Blue = 4:1	Photoreceptor-mediated light signaling regulates PAL, CHS, CHI, and FLS in the phenylpropanoid–flavonoid pathway, promoting the accumulation of flavonoids, anthocyanins, and kinsenoside.	[108,109]

## Data Availability

No new data was generated for this review article.

## Supplementary Materials

The following supporting information can be downloaded at: https://www.mdpi.com/article/10.3390/metabo16070479/s1, The full database search formulas for all retrieved databases are provided in Supplementary File S1. Supplementary File S1: Search strategies and eligibility criteria. Supplementary File S2: PRISMA checklist.

## References

[1] Elshafie H.S., Camele I., Mohamed A.A. (2023). A Comprehensive Review on the Biological, Agricultural and Pharmaceutical Properties of Secondary Metabolites Based-Plant Origin. Int. J. Mol. Sci..

[2] Paik I., Huq E. (2019). Plant photoreceptors: Multi-functional sensory proteins and their signaling networks. Semin. Cell Dev. Biol..

[3] Agati G., Azzarello E., Pollastri S., Tattini M. (2012). Flavonoids as antioxidants in plants: Location and functional significance. Plant Sci..

[4] Givnish T. (1988). Adaptation to Sun and Shade: A Whole-Plant Perspective. Aust. J. Plant Physiol..

[5] Chen M., Chory J., Fankhauser C. (2004). Light Signal Transduction in Higher Plants. Annu. Rev. Genet..

[6] Gangappa S.N., Botto J.F. (2016). The Multifaceted Roles of HY5 in Plant Growth and Development. Mol. Plant.

[7] Lingwan M., Pradhan A.A., Kushwaha A.K., Dar M.A., Bhagavatula L., Datta S. (2023). Photoprotective Role of Plant Secondary Metabolites: Biosynthesis, Photoregulation, and Prospects of Metabolic Engineering for Enhanced Protection Under Excessive Light. Environ. Exp. Bot..

[8] Leivar P., Tepperman J.M., Cohn M.M., Monte E., Al-Sady B., Erickson E., Quail P.H. (2012). Dynamic Antagonism between Phytochromes and PIF Family Basic Helix-Loop-Helix Factors Induces Selective Reciprocal Responses to Light and Shade in a Rapidly Responsive Transcriptional Network in *Arabidopsis*. Plant Cell.

[9] Lv Z.-Y., Sun W.-J., Jiang R., Chen J.-F., Ying X., Zhang L., Chen W.-S. (2021). Phytohormones Jasmonic Acid, Salicylic Acid, Gibberellins, and Abscisic Acid are Key Mediators of Plant Secondary Metabolites. World J. Tradit. Chin. Med..

[10] Wu W., Wu H., Liang R., Huang S., Meng L., Zhang M., Xie F., Zhu H. (2025). Light Regulates the Synthesis and Accumulation of Plant Secondary Metabolites. Front. Plant Sci..

[11] Zhang S., Ma J., Zou H., Zhang L., Li S., Wang Y. (2020). The Combination of Blue and Red LED Light Improves Growth and Phenolic Acid Contents in *Salvia miltiorrhiza* Bunge. Ind. Crops Prod..

[12] Zhang T., Zhang Y.-H., Yang J.-X., Wang X.-Z., Yang Q.-Q., Zhu X.-J., Cao X.-Y. (2022). Transcriptome and Targeted Metabolome Analysis Revealed the Effects of Combined Red and Blue Light on the Growth and Secondary Metabolism of *Scutellaria baicalensis* Georgi. Ind. Crops Prod..

[13] Yu L., Bu L., Li D., Zhu K., Zhang Y., Wu S., Chang L., Ding X., Jiang Y. (2024). Effects of Far-Red Light and Ultraviolet Light-A on Growth, Photosynthesis, Transcriptome, and Metabolome of Mint (*Mentha haplocalyx* Briq.). Plants.

[14] Page M.J., McKenzie J.E., Bossuyt P.M., Boutron I., Hoffmann T.C., Mulrow C.D., Shamseer L., Tetzlaff J.M., Akl E.A., Brennan S.E. (2021). The PRISMA 2020 Statement: An Updated Guideline for Reporting Systematic Reviews. BMJ.

[15] Rockwell N.C., Su Y.-S., Lagarias J.C. (2006). Phytochrome Structure and Signaling Mechanisms. Annu. Rev. Plant Biol..

[16] Burgie E.S., Zhang J., Vierstra R.D. (2016). Crystal Structure of Deinococcus Phytochrome in the Photoactivated State Reveals a Cascade of Structural Rearrangements during Photoconversion. Structure.

[17] Favory J.-J., Stec A., Gruber H., Rizzini L., Oravecz A., Funk M., Albert A., Cloix C., Jenkins G.I., Oakeley E.J. (2009). Interaction of COP1 and UVR8 regulates UV-B-induced photomorphogenesis and stress acclimation in *Arabidopsis*. EMBO J..

[18] Jenkins G.I. (2014). The UV-B Photoreceptor UVR8: From Structure to Physiology. Plant Cell..

[19] Osterlund M.T., Hardtke C.S., Wei N., Deng X.W. (2000). Targeted Destabilization of HY5 During Light-Regulated Development of *Arabidopsis*. Nature.

[20] Lau O.S., Deng X.W. (2012). The Photomorphogenic Repressors COP1 and DET1, 20 Years Later. Trends Plant Sci..

[21] Xiao Y., Chu L., Zhang Y., Bian Y., Xiao J., Xu D. (2022). HY5, A Pivotal Regulator of Light-Dependent Development in Higher Plants. Front. Plant Sci..

[22] Lee J., He K., Stolc V., Lee H., Figueroa P., Gao Y., Tongprasit W., Zhao H., Lee I., Deng X.W. (2007). Analysis of Transcription Factor HY5 Genomic Binding Sites Revealed Its Hierarchical Role in Light Regulation of Development. Plant Cell.

[23] Qi J., Zhang M., Lu C., Hettenhausen C., Tan Q., Cao G., Zhu X., Wu G., Wu J. (2018). Ultraviolet-B Enhances the Resistance of Multiple Plant Species to Lepidopteran Insect Herbivory Through the Jasmonic Acid Pathway. Sci. Rep..

[24] Kazan K., Manners J.M. (2013). MYC2, The Master in Action. Mol. Plant.

[25] Nuerlan K., Li Y., Zhang J., Guo J., Ma X., Wang Y., Chen K., Hu Y., Tong Y. (2025). *CYP*450: A Crucial Player in Active Ingredient Biosynthesis in Medicinal Plants. Sci. Tradit. Chin. Med..

[26] Kim Y.-H., Park C.-W., Lee K.M., Hong C.-O., Son H.-J., Kim K.K., Park H.C., Kim Y.-J. (2025). The Roles of MYC2 Transcription Factor in JA-Signaling Pathway in Plants. J. Plant Biol..

[27] Hou X., Lee L.Y.C., Xia K., Yan Y., Yu H. (2010). DELLAs Modulate Jasmonate Signaling via Competitive Binding to JAZs. Dev. Cell..

[28] Yu Z.-X., Li J.-X., Yang C.-Q., Hu W.-L., Wang L.-J., Chen X.-Y. (2012). The Jasmonate-Responsive AP2/ERF Transcription Factors AaERF1 and AaERF2 Positively Regulate Artemisinin Biosynthesis in *Artemisia annua* L.. Mol. Plant.

[29] Ballaré C.L., Pierik R. (2017). The Shade-Avoidance Syndrome: Multiple Signals and Ecological Consequences. Plant Cell Environ..

[30] Genoud T., Buchala A.J., Chua N.-H., Métraux J.-P. (2002). Phytochrome Signalling Modulates the SA-Perceptive Pathway in *Arabidopsis*. Plant J..

[31] Vlot A.C., Dempsey D.A., Klessig D.F. (2009). Salicylic Acid, a Multifaceted Hormone to Combat Disease. Annu. Rev. Phytopathol..

[32] Davière J.-M., Achard P. (2013). Gibberellin Signaling in Plants. Development.

[33] Ballaré C.L. (2014). Light Regulation of Plant Defense. Annu. Rev. Plant Biol..

[34] Dodd A.N., Salathia N., Hall A., Kévei E., Tóth R., Nagy F., Hibberd J.M., Millar A.J., Webb A.R. (2005). Plant Circadian Clocks Increase Photosynthesis, Growth, Survival, and Competitive Advantage. Science.

[35] Leivar P., Quail P.H. (2011). PIFs: Pivotal Components in a Cellular Signaling Hub. Trends Plant Sci..

[36] Wu D., Liu M., Yu W., Cui M., Huang X., Ning F., Chingin K., Luo L. (2022). Red:Blue LED Light Proportion Affects Biomass Accumulation and Polyamine Metabolism in *Anoectochilus roxburghii* Studied by Nano-Electrospray Ionization Mass Spectrometry. Ind. Crops Prod..

[37] Di P., Yang X., Wan M., Han M., Zhang Y., Yang L. (2023). Integrative Metabolomic and Transcriptomic Reveals Potential Mechanism for Promotion of Ginsenoside Synthesis in *Panax Ginseng* Leaves under Different Light Intensities. Front. Bioeng. Biotechnol..

[38] Vázquez-Flotaand F.A., Luca V.D. (1998). Jasmonate Modulates Development- and Light-Regulated Alkaloid Biosynthesis in *Catharanthus roseus* Fn1. In honour of Professor G. H. Neil Towers 75th Birthday. Phytochemistry.

[39] Yeşil M., Özcan M.M. (2021). Effects of Harvest Stage and Diurnal Variability on Yield and Essential Oil Content in *Mentha* × *Piperita* L.. Plant Soil Environ..

[40] Müller P., Li X.-P., Niyogi K.K. (2001). Non-Photochemical Quenching. A Response to Excess Light Energy. Plant Physiol..

[41] Creux N., Harmer S. (2019). Circadian Rhythms in Plants. Cold Spring Harb. Perspect. Biol..

[42] Venkat A., Muneer S. (2022). Role of Circadian Rhythms in Major Plant Metabolic and Signaling Pathways. Front. Plant Sci..

[43] Asada K. (2006). Production and Scavenging of Reactive Oxygen Species in Chloroplasts and Their Functions. Plant Physiol..

[44] Niyogi K.K. (1999). Photoprotection Revisited: Genetic and Molecular Approaches. Annu. Rev. Plant. Biol..

[45] Winkel-Shirley B. (2001). Flavonoid Biosynthesis. A Colorful Model for Genetics, Biochemistry, Cell Biology, and Biotechnology. Plant Physiol..

[46] Ferreyra M.L.F., Serra P., Casati P. (2021). Recent Advances on the Roles of Flavonoids as Plant Protective Molecules after UV and High Light Exposure. Physiol. Plant..

[47] Apel K., Hirt H. (2004). Reactive Oxygen Species: Metabolism, Oxidative Stress, and Signal Transduction. Annu. Rev. Plant Biol..

[48] Fang H., Guo C., Mei X., Hao M., Zhang J., Luo L., Liu H., Liu Y., Huang H., He X. (2024). Light Stress Elicits Soilborne Disease Suppression Mediated by Root-Secreted Flavonoids in *Panax notoginseng*. Hortic. Res..

[49] Pietta P.-G. (2000). Flavonoids as Antioxidants. J. Nat. Prod..

[50] Anjali, Kumar S., Korra T., Thakur R., Arutselvan R., Kashyap A.S., Nehela Y., Chaplygin V., Minkina T., Keswani C. (2023). Role of Plant Secondary Metabolites in Defence and Transcriptional Regulation in Response to Biotic Stress. Plant Stress..

[51] Zhang C., Wang S., Li Q., Zhang Y., He Y., Yan B., Zhou L., Guo L. (2025). Metabolomic Profiling and Chemical Marker Identification in Medicinal Plants of *Atractylodes*. Sci. Tradit. Chin. Med..

[52] Fang H., Qi X., Li Y., Yu X., Xu D., Liang C., Li W., Liu X. (2020). De Novo Transcriptomic Analysis of Light-Induced Flavonoid Pathway, Transcription Factors in the Flower Buds of *Lonicera japonica*. Trees.

[53] Fang H., Wan Y., Liu H., Qi X., Yu X., Chen Z., Liu Q., Li L., Bai Y., Liu D. (2025). Characterization of the *Lonicera japonica* R2R3-MYB Transcription Factor Gene LjaMYB305 That Promotes the Flavonoid Biosynthesis. Plant Sci..

[54] Xu Y., Wang G., Cao F., Zhu C., Wang G., El-Kassaby Y.A. (2014). Light Intensity Affects the Growth and Flavonol Biosynthesis of Ginkgo (*Ginkgo biloba* L.). New For..

[55] Zhang X., Ding X., Ji Y., Wang S., Chen Y., Luo J., Shen Y., Peng L. (2018). Measurement of Metabolite Variations and Analysis of Related Gene Expression in Chinese Liquorice (*Glycyrrhiza uralensis*) Plants under UV-B Irradiation. Sci. Rep..

[56] Zhong C., Chen C., Gao X., Tan C., Bai H., Ning K. (2022). Multi-omics Profiling Reveals Comprehensive Microbe–Plant–Metabolite Regulation Patterns for Medicinal Plant *Glycyrrhiza uralensis* Fisch. Plant Biotechnol. J..

[57] Jiang Y., Zhang Z., Zhang S., Chen X., Li B., Ma S., Wang Y., Sun Z. (2025). Transcriptome and Metabolomics Analysis Reveal the Effects of Red and Blue Light on the Physiology and Primary Medicinal Components (Liquiritin and Glycyrrhizic Acid) of *Glycyrrhiza uralensis* Seedlings. Int. J. Mol. Sci..

[58] Ren C., Wang J., Xian B., Tang X., Liu X., Hu X., Hu Z., Wu Y., Chen C., Wu Q. (2020). Transcriptome Analysis of Flavonoid Biosynthesis in Safflower Flowers Grown under Different Light Intensities. PeerJ.

[59] Cheng J., Guo F., Liang W., Wang H., Chen Y., Dong P. (2025). Callus Culture System from *Lonicera japonica* Thunb Anthers: Light Quality Effects on Callus Quality Evaluation. Int. J. Mol. Sci..

[60] Zhong J., Seki T., Kinoshita S., Yoshida T. (1991). Effect of Light Irradiation on Anthocyanin Production by Suspended Culture of *Perilla frutescens*. Biotechnol. Bioeng..

[61] Raffo A., Mozzanini E., Ferrari Nicoli S., Lupotto E., Cervelli C. (2020). Effect of Light Intensity and Water Availability on Plant Growth, Essential Oil Production and Composition in *Rosmarinus officinalis* L.. Eur. Food Res. Technol..

[62] Liu X.-M., Tan J.-P., Cheng S.-Y., Chen Z.-X., Ye J.-B., Zheng J.-R., Xu F., Zhang W.-W., Liao Y.-L., Yang X.-Y. (2022). Comparative Transcriptome Analysis Provides Novel Insights into the Molecular Mechanism of Berberine Biosynthesis in *Coptis chinensis*. Sci. Hortic..

[63] Ke W., Li Y., Zhong F., Pen M., Dong J., Xu B., Ma Y., Zhou T. (2023). Relatively High Light Inhibits Reserves Degradation in the *Coptis chinensis* Rhizome during the Leaf Expansion by Changing the Source-Sink Relationship. Front. Plant Sci..

[64] Liang Y., Wang L. (2022). *Carthamus tinctorius* L.: A Natural Neuroprotective Source for Anti-Alzheimer’s Disease Drugs. J. Ethnopharmacol..

[65] Xian B., Wang R., Jiang H., Zhou Y., Yan J., Huang X., Chen J., Wu Q., Chen C., Xi Z. (2022). Comprehensive Review of Two Groups of Flavonoids in *Carthamus tinctorius* L.. Biomed. Pharmacother..

[66] Ahmad N., Li T., Liu Y., Hoang N.Q.V., Ma X., Zhang X., Liu J., Yao N., Liu X., Li H. (2020). Molecular and Biochemical Rhythms in Dihydroflavonol 4-Reductase- Mediated Regulation of Leucoanthocyanidin Biosynthesis in *Carthamus tinctorius* L.. Ind. Crops Prod..

[67] Singh D., Basu C., Meinhardt-Wollweber M., Roth B. (2015). LEDs for Energy Efficient Greenhouse Lighting. Renew. Sustain. Energy Rev..

[68] Fan S., Ahmad N., Libo J., Xinyue Z., Xintong M., Hoang N.Q.V., Mallano A.I., Nan W., Zhuoda Y., Xiuming L. (2021). Genome-Wide Investigation of Hydroxycinnamoyl CoA: Shikimate Hydroxycinnamoyl Transferase (HCT) Gene Family in *Carthamus tinctorius* L.. Not. Bot. Horti Agrobot..

[69] Yang Z., Gao D., Wang Y., Liu H., Wu Y., Zhang H., Wang H., Gao X., Wang J., Wang Y. (2026). Physiological and Multi-Omics Insights into Ultraviolet B-Induced Stress Adaptation in *Fritillaria Cirrhosa Native* to the Qinghai-Tibet Plateau. J. Adv. Res..

[70] Franklin K.A., Whitelam G.C. (2005). Phytochromes and Shade-Avoidance Responses in Plants. Ann. Bot..

[71] Leivar P., Monte E. (2014). PIFs: Systems Integrators in Plant Development. Plant Cell..

[72] Chico J.-M., Fernández-Barbero G., Chini A., Fernández-Calvo P., Díez-Díaz M., Solano R. (2014). Repression of Jasmonate-Dependent Defenses by Shade Involves Differential Regulation of Protein Stability of MYC Transcription Factors and Their JAZ Repressors in Arabidopsis. Plant Cell..

[73] Di P., Sun Z., Cheng L., Han M., Yang L., Yang L. (2023). LED Light Irradiations Differentially Affect the Physiological Charac-teristics, Ginsenoside Content, and Expressions of Ginsenoside Biosynthetic Pathway Genes in *Panax ginseng*. Agriculture.

[74] Dai C., Lin Y., Guan J., Meng T., Liu Y., Cui X., Guo L., Yang Y. (2024). Mechanism Analysis: Nitrogen and Potassium Synergy Regulate Nitrogen Distribution in Photosynthetic System to Enhance *Panax notoginseng* Resistance to Light Stress. Ind. Crops Prod..

[75] Gao L.-L., Dong Y., Cun Z., Zhang J.-Y., Chen J.-W. (2025). Moderate Shading Elicits Succinic Acid Accumulation Aligning with Simultaneous Expression of Genes Involved in TCA Cycle and Photosynthetic Pathway in a Medicinal Plant *Pinellia ternata*. Plant Physiol. Biochem..

[76] Chen Q., Wu X., Qu Y., Li N., Cui X., Ge F. (2026). PnWRKY38-PnSUS1 Axis Regulates the Biosynthesis of *Panax notoginseng* Saponins. Hortic. Res..

[77] Shuang S.P., Zhang J.Y., Cun Z., Wu H.M., Meng Z.G. (2022). Ecophysiological Characteristics of a Typically Shade-Tolerant Species *Panax notoginseng* in Response to Different Light Intensities. Acta Ecol. Sin..

[78] Liu Z.-Q., Wang Y., Wang X., Peng N., Yang S.-S., Shao H.-H., Jiao X.-L., Gao W.-W. (2022). Effect of Light Intensity on Growth, Accumulation of Ginsenosides, and Expression of Related Enzyme Genes of *Panax quinquefolius*. J. Tradit. Chin. Med..

[79] Wang Z., Ma H., Zhang M., Wang Z., Tian Y., Li W., Wang Y. (2021). Transcriptional Response of *Asarum heterotropoides* Fr. Schmidt var. *Mandshuricum* (Maxim.) Kitag. Leaves Grown Under Full and Partial Daylight Conditions. BMC Genom..

[80] Wang Z., Xiao S., Wang Y., Liu J., Ma H., Wang Y., Tian Y., Hou W. (2020). Effects of Light Irradiation on Essential Oil Biosynthesis in the Medicinal Plant *Asarum heterotropoides* Fr. Schmidt Var. *Mandshuricum* (Maxim) Kitag. PLoS ONE.

[81] Nurcholis W., Rahmadansah R., Astuti P., Priosoeryanto B.P., Arianti R., Kristóf E. (2024). Comparative Analysis of Volatile Compounds and Biochemical Activity of *Curcuma Xanthorrhiza Roxb*. Essential Oil Extracted from Distinct. Shaded Plants. Plants.

[82] Rizzini L., Favory J.-J., Cloix C., Faggionato D., O’Hara A., Kaiserli E., Baumeister R., Schäfer E., Nagy F., Jenkins G.I. (2011). Perception of UV-B by the Arabidopsis UVR8 Protein. Science.

[83] Stracke R., Ishihara H., Huep G., Barsch A., Mehrtens F., Niehaus K., Weisshaar B. (2007). Differential Regulation of Closely Related R2R3-MYB Transcription Factors Controls Flavonol Accumulation in Different Parts of the Arabidopsis Thaliana Seedling. Plant J..

[84] Yin X., Fan H., Chen Y., Li L., Song W., Fan Y., Zhou W., Ma G., Alolga R.N., Li W. (2020). Integrative Omic and Transgenic Analyses Reveal the Positive Effect of ultraviolet-B Irradiation on Salvianolic Acid Biosynthesis through Upregulation of SmNAC1. Plant J..

[85] Yu Y., Jiang W., Chen S., Yin W., Hao P., Zhu Z., Mahmood M.S., Abbas R.Z., Mehmood K., Zhou Q. (2025). A Study on *Glycyrrhiza uralensis* Polysaccharide: The Structure Elucidation, Anti-Inflammation Effects and Treatment Mechanism of LPS-Induced Pneumonia Mice. Int. J. Biol. Macromol..

[86] Liu J.-L., Shu Z.-M., Liang Z., Shi X., Zhang Y. (2013). UV-B Radiation Effects on Phenolic Changes and Antioxidant Activity in *Salvia miltiorrhiza* Bunge Leaf. J. Food Agric. Environ..

[87] Zhong Z., Liu S., Han S., Li Y., Tao M., Liu A., He Q., Chen S., Dufresne C., Zhu W. (2021). Integrative Omic Analysis Reveals the Improvement of Alkaloid Accumulation by Ultraviolet-B Radiation and Its Upstream Regulation in *Catharanthus roseus*. Ind. Crops Prod..

[88] Rady M.R., Gierczik K., Ibrahem M.M., Matter M.A., Galiba G. (2021). Anticancer Compounds Production in *Catharanthus roseus* by Methyl Jasmonate and UV-B Elicitation. S. Afr. J. Bot..

[89] Zhang D., Sun W., Shi Y., Wu L., Zhang T., Xiang L. (2018). Red and Blue Light Promote the Accumulation of Artemisinin in *Artemisia annua* L.. Molecules.

[90] Lopes E.M., Guimarães-Dias F., Gama T.D.S.S., Macedo A.L., Valverde A.L., De Moraes M.C., De Aguiar-Dias A.C.A., Bizzo H.R., Alves-Ferreira M., Tavares E.S. (2020). *Artemisia annua* L. and Photoresponse: From Artemisinin Accumulation, Volatile Profile and Anatomical Modifications to Gene Expression. Plant Cell. Rep..

[91] Fu X., He Y., Li L., Zhao L., Wang Y., Qian H., Sun X., Tang K., Zhao J. (2021). Overexpression of Blue Light Receptor AaCRY1 Improves Artemisinin Content in *Artemisia annua* L.. Biotech. App Biochem..

[92] An X., Liao Y., Yu Y., Fan J., Wan J., Wei Y., Ouyang Z. (2024). Effects of MhMYB1 and MhMYB2 Transcription Factors on the Monoterpenoid Biosynthesis Pathway in L-Menthol Chemotype of *Mentha haplocalyx* Briq. Planta.

[93] Wei G., Zhang G., Li M., Zheng Y., Zheng W., Wang B., Zhang Z., Zhang X., Huang Z., Wei T. (2024). *Panax notoginseng*: Panoramagram of Phytochemical and Pharmacological Properties, Biosynthesis, and Regulation and Production of Ginsenosides. Hortic. Res..

[94] Yu M., Ma C., Tai B., Fu X., Liu Q., Zhang G., Zhou X., Du L., Jin Y., Han Y. (2025). Unveiling the Regulatory Mechanisms of Nodules Development and Quality Formation in *Panax notoginseng* Using Multi-Omics and MALDI-MSI. J. Adv. Res..

[95] Zhang H., Hedhili S., Montiel G., Zhang Y., Chatel G., Pré M., Gantet P., Memelink J. (2011). The Basic Helix-Loop-Helix Tran-scription Factor CrMYC2 Controls the Jasmonate-responsive Expression of the ORCA Genes That Regulate Alkaloid Biosynthesis in *Catharanthus roseus*. Plant J..

[96] Chang C., Guo X., Wang B., Wang Y., Zhang M., Yu F., Tang Z. (2025). CrGRF1/4 Mediating Light Signal to Regulate Monoterpenoid Indole Alkaloid Biosynthesis in *Catharanthus roseus*. Plant Cell. Environ..

[97] Li X., Yu H., Guo Y., Liu J., Yu W., Guo X. (2025). Comparative Role of Oxidative Balance, Alkaloid Production, and Metabolites in Exposure to Red-to-Blue Light in *Catharanthus roseus*. Plant Physiol. Biochem..

[98] Wang Z., Wang G., Quan X., Zhang M., Wang Y., Cui L., Li H., Zhang Z., Hou W. (2024). Effect of Different Light Qualities on Essential Oil and Asarinin in *Asarum heterotropoides* Fr. Schmidt Var. *Mandshuricum* (Maxim.) Kitag. Horticulturae.

[99] Ma D., Pu G., Lei C., Ma L., Wang H., Guo Y., Chen J., Du Z., Wang H., Li G. (2009). Isolation and Characterization of AaWRKY1, an *Artemisia annua* Transcription Factor That Regulates the Amorpha-4,11-Diene Synthase Gene, a Key Gene of Artemisinin Biosynthesis. Plant Cell. Physiol..

[100] Hao X., Zhong Y., Nötzmann H.-W., Fu X., Yan T., Shen Q., Chen M., Ma Y., Zhao J., Osbourn A. (2019). Light-Induced Artemisinin Biosynthesis Is Regulated by the bZIP Transcription Factor AaHY5 in *Artemisia annua*. Plant Cell Physiol..

[101] Wang Y., Zhang H., Zhao B., Yuan X. (2001). Improved Growth of *Artemisia annua* L. Hairy Roots and Artemisinin Production under Red Light Conditions. Biotechnol. Lett..

[102] Su P., Ding S., Wang D., Kan W., Yuan M., Chen X., Tang C., Hou J., Wu L. (2024). Plant Morphology, Secondary Metabolites and Chlorophyll Fluorescence of *Artemisia argyi* under Different LED Environments. Photosynth. Res..

[103] Yang Y., Tong Y.X. (2025). Effects of Different Green-Blue Light Ratios on Growth, Stomatal Characteristics, and Water Use Efficiency of Basil Plants (*Ocimum basilicum* L.) in a Plant Factory. Acta Hortic..

[104] Seo J.W., Choi H.J., Park J., Choi W.H., Seong E.S. (2025). Transcriptome-Based Identification of MYB Transcription Factors Associated with Flavonoid Biosynthesis under LED Light in *Astragalus membranaceus* (Fisch.) Bunge. Not. Bot. Horti. Agrobo..

[105] Seo J.W., Lee J.G., Yoo J.H., Lim J.D., Choi I.Y., Kim M.J., Yu C.Y., Seong E.S. (2023). Cellular Morphology and Transcriptome Comparative Analysis of *Astragalus membranaceus* Bunge Sprouts Cultured In Vitro under Different LED Light. Plants.

[106] Fang S., Qiu S., Chen K., Lv Z., Chen W. (2023). The Transcription Factors SbMYB45 and SbMYB86.1 Regulate Flavone Biosynthesis in *Scutellaria baicalensis*. Plant Physiol. Biochem..

[107] Mulas G., Gardner Z., Craker L.E. (2006). Effect of Light Quality on Growth and Essential Oil Composition in Rosemary. Acta Hortic..

[108] Cao J., Zeng J., Hu R., Liang W., Zheng T., Yang J., Liang X., Huang X., Chen Y. (2024). Comparative Metabolome and Transcriptome Analyses of the Regulatory Mechanism of Light Intensity in the Synthesis of Endogenous Hormones and Antho-cyanins in *Anoectochilus roxburghii* (Wall.) Lindl. Genes.

[109] Gam D.T., Khoi P.H., Ngoc P.B., Linh L.K., Hung N.K., Anh P.T.L., Thu N.T., Hien N.T.T., Khanh T.D., Ha C.H. (2020). LED Lights Promote Growth and Flavonoid Accumulation of *Anoectochilus roxburghii* and Are Linked to the Enhanced Expression of Several Related Genes. Plants.

[110] Gao C. (2021). Genome Engineering for Crop Improvement and Future Agriculture. Cell.

[111] Li R., Wang J., Han Y., Dai Z. (2024). Compound Danshen Yeast 1.0. Sci. Tradit. Chin. Med..

[112] Valladares F., Niinemets Ü. (2008). Shade Tolerance, a Key Plant Feature of Complex Nature and Consequences. Annu. Rev. Ecol. Evol. Syst..

[113] Huang L., Guo L., Zhang X., Yu L., Sun J. (2024). Species of Chinese Materia Medica Resources Based on the Fourth National Survey of Chinese Materia Medica Resources. Sci. Tradit. Chin. Med..

