# Supplementary material for: Integrative Perspectives on Light-Regulated Metabolism in Medicinal Plants
Source: Metabolites. 2026 Jul 8;16(7):479. doi: 10.3390/metabo16070479 (PMC13413876; doi:10.3390/metabo16070479)
Supplement: Supplementary file 1 [file metabolites-16-00479-s001.zip › Supplementary File S1.pdf]

SUPPLEMENTARY MATERIAL

Supplementary File S1. Search strategies and eligibility criteria

Siqian Xiao[1,3], Dan Gao[1,*], Tielin Wang[2], Binbin Yan[2], Feng Xiong[2], Chongning Lv[3,*], Chuanzhi Kang[1]

[1] State Key Laboratory for Quality Ensurance and Sustainable Use of Dao-di Herbs, Institute of Chinese Materia Medica, China Academy of Chinese Medical Sciences, Beijing 100700, China

[2] National Resource Center for Chinese Materia Medica, China Academy of Chinese Medical Sciences, Beijing, 100700, PR China

[3] College of Chinese Materia Medica, Shenyang Pharmaceutical University, Liaoning 110016, China

[*] Corresponding author: Chongning Lv, Dan Gao and Chuanzhi Kang. E-mail:lcnmi@syphu.edu.cn； dgao@icmm.ac.cn; zystny01@126.com

Table S1. Database search strategies used for the systematic review.

**Database**	**Search period**	**Language restriction**	**Document types**	**Search strategy**
Web of Science Core Collection	Database inception to 31 May 2026	English	Original research articles and dissertations containing original experimental data; reviews were used only for reference tracing and were not counted as included studies.	TS = (<br>(light OR "light intensity" OR "light quality" OR "spectral quality" OR photoperiod OR LED OR "red light" OR "blue light" OR "white light" OR "green light" OR "far-red light" OR UV OR UV-A OR UV-B OR phytochrome* OR cryptochrome* OR phototropin* OR UVR8 OR COP1 OR HY5)<br>AND ("secondary metabolism" OR "secondary metabolite*" OR phytochemical* OR "bioactive compound*" OR phenolic* OR flavonoid* OR terpenoid* OR alkaloid* OR saponin* OR "essential oil*")<br>AND ("medicinal plant*" OR "medicinal herb*" OR "herbal medicine*" OR "Chinese medicinal plant*" OR "traditional Chinese medicine" OR "medicinal crop*")<br>)
Scopus	Database inception to 31 May 2026	English	Original research articles and dissertations containing original experimental data; reviews were used only for reference tracing and were not counted as included studies.	TITLE-ABS-KEY (<br>(light OR "light intensity" OR "light quality" OR "spectral quality" OR photoperiod OR LED OR "red light" OR "blue light" OR "white light" OR "green light" OR "far-red light" OR UV OR UV-A OR UV-B OR phytochrome* OR cryptochrome* OR phototropin* OR UVR8 OR COP1 OR HY5)<br>AND ("secondary metabolism" OR "secondary metabolite*" OR phytochemical* OR "bioactive compound*" OR phenolic* OR flavonoid* OR terpenoid* OR alkaloid* OR saponin* OR "essential oil*")<br>AND ("medicinal plant*" OR "medicinal herb*" OR "herbal medicine*" OR "Chinese medicinal plant*" OR "traditional Chinese medicine" OR "medicinal crop*")<br>)

Database	Search period	Language restriction	Document types	Search strategy
PubMed	Database inception to 31 May 2026	English	Original research articles containing original experimental data; reviews were used only for reference tracing and were not counted as included studies.	((light[Title/Abstract] OR "light intensity"[Title/Abstract] OR "light quality"[Title/Abstract] OR "spectral quality"[Title/Abstract] OR photoperiod[Title/Abstract] OR LED[Title/Abstract] OR "red light"[Title/Abstract] OR "blue light"[Title/Abstract] OR "white light"[Title/Abstract] OR "green light"[Title/Abstract] OR "far-red light"[Title/Abstract] OR UV[Title/Abstract] OR UV-A[Title/Abstract] OR UV-B[Title/Abstract] OR phytochrome*[Title/Abstract] OR cryptochrome*[Title/Abstract] OR phototropin*[Title/Abstract] OR UVR8[Title/Abstract] OR COP1[Title/Abstract] OR HY5[Title/Abstract]) AND ("secondary metabolism"[Title/Abstract] OR "secondary metabolite"[Title/Abstract] OR "secondary metabolites"[Title/Abstract] OR phytochemical*[Title/Abstract] OR "bioactive compound"[Title/Abstract] OR "bioactive compounds"[Title/Abstract] OR phenolic*[Title/Abstract] OR flavonoid*[Title/Abstract] OR terpenoid*[Title/Abstract] OR alkaloid*[Title/Abstract] OR saponin*[Title/Abstract] OR "essential oil"[Title/Abstract] OR "essential oils"[Title/Abstract]) AND ("medicinal plant"[Title/Abstract] OR "medicinal plants"[Title/Abstract] OR "medicinal herb"[Title/Abstract] OR "medicinal herbs"[Title/Abstract] OR "herbal medicine"[Title/Abstract] OR "Chinese medicinal plant"[Title/Abstract] OR "Chinese medicinal plants"[Title/Abstract] OR "traditional Chinese medicine"[Title/Abstract] OR "medicinal crop"[Title/Abstract] OR "medicinal crops"[Title/Abstract]))

Additional manual search: The reference lists of highly cited reviews and key original studies were manually screened to identify additional eligible original experimental studies. Reviews and systematic reviews were used only for citation tracing and were not included in the evidence synthesis unless they contained original experimental data that met all eligibility criteria.

Eligibility criteria

Included:

1. English-language original experimental studies on medicinal plants or medicinal herbs.
2. Studies applying defined light treatments, including light intensity, spectral quality, photoperiod, UV irradiation, LED lighting, shading, or red/blue/far-red light treatments.
3. Studies reporting at least one outcome related to secondary metabolism, including metabolite accumulation or composition, relevant gene expression, transcriptomic or metabolomic data, enzyme activity, or functional validation.
4. Studies providing sufficient methodological information on plant material, light treatment conditions, controls or comparison groups, and measured outcomes.
5. Dissertations were considered only when they contained original experimental data and met all criteria above.

Excluded:

1. Conference abstracts, editorials, letters, protocols, and short communications without sufficient experimental data.
2. Reviews, systematic reviews, and meta-analyses, except when used for reference tracing.
3. Duplicate publications; when duplicate records were identified, the most complete or most recent report was retained.
4. Studies without a valid light-treatment experiment, without a control or comparison group, or without sufficient methodological detail.
5. Studies not focused on medicinal plants or not reporting outcomes related to secondary metabolism.
6. Non-English publications for which the full text could not be reliably assessed.
