# Supplementary material for: Integrative Perspectives on Light-Regulated Metabolism in Medicinal Plants
Source: Metabolites. 2026 Jul 8;16(7):479. doi: 10.3390/metabo16070479 (PMC13413876; doi:10.3390/metabo16070479)
Supplement: Supplementary file 1 [file metabolites-16-00479-s001.zip › Supplementary File S2. PRISMA Checklist.pdf]

PRISMA 2020 Main Checklist

Topic	No.	Item	Location where item is reported
**TITLE**			
**Title**	1	Identify the report as a systematic review.	Page 1, The title clearly identifies this paper as a systematic review.
**ABSTRACT**			
**Abstract**	2	See the PRISMA 2020 for Abstracts checklist	
**INTRODUCTION**			
**Rationale**	3	Describe the rationale for the review in the context of existing knowledge.	Page 1, Abstract elaborates the research gap and core review objectives.
**Objectives**	4	Provide an explicit statement of the objective(s) or question(s) the review addresses.	Page 2 Introduction, explicitly states the review’s research question on light-mediated secondary metabolism in medicinal plants.
**METHODS**			
**Eligibility criteria**	5	Specify the inclusion and exclusion criteria for the review and how studies were grouped for the syntheses.	Page 2 Methods, clear inclusion/exclusion criteria for light-treatment original experiments are fully defined.
**Information sources**	6	Specify all databases, registers, websites, organisations, reference lists and other sources searched or consulted to identify studies. Specify the date when each source was last searched or consulted.	Page 2 Methods, databases Web of Science, Scopus, PubMed were searched up to May 2026.

Topic	No.	Item	Location where item is reported
**Search strategy**	7	Present the full search strategies for all databases, registers and websites, including any filters and limits used.	Supplementary File 1, full complete search formulas for all databases are attached.
**Selection process**	8	Specify the methods used to decide whether a study met the inclusion criteria of the review, including how many reviewers screened each record and each report retrieved, whether they worked independently, and if applicable, details of automation tools used in the process.	Page 3 Methods and Figure 1. Two reviewers independently screened titles and abstracts, followed by full–text assessment according to the predefined inclusion and exclusion criteria. Disagreements were resolved through discussion until consensus was reached. No automated screening tools were used.
**Data collection process**	9	Specify the methods used to collect data from reports, including how many reviewers collected data from each report, whether they worked independently, any processes for obtaining or confirming data from study investigators, and if applicable, details of automation tools used in the process.	Page 3Methods. Data were independently extracted by two reviewers using a predesigned standardized extraction form. Extracted information included plant species, light treatment conditions,target secondary metabolites, gene expression indicators, and key experimental characteristics.Discrepancies were checked and resolved by discussion. Missing or unclear information was recorded as not reported. No study investigators were contacted, and no automated data extraction tools were used.
**Data items**	10a	List and define all outcomes for which data were sought. Specify whether all results that were compatible with each outcome domain in each study were sought (e.g. for all measures, time points, analyses), and if not, the methods used to decide which results to collect.	Page 3 Methods, all target secondary metabolites and gene expression indicators are defined as core research outcomes.

Topic	No.	Item	Location where item is reported
	10b	List and define all other variables for which data were sought (e.g. participant and intervention characteristics, funding sources). Describe any assumptions made about any missing or unclear information.	Page 3 Methods, plant species, light treatment conditions and basic study information were extracted as auxiliary variables.
**Study risk of bias assessment**	11	Specify the methods used to assess risk of bias in the included studies, including details of the tool(s) used, how many reviewers assessed each study and whether they worked independently, and if applicable, details of automation tools used in the process.	Page 3 Methods, two reviewers independently assessed experimental reproducibility narratively; clinical bias assessment tools were not applicable. Risk of bias was narratively assessed based on experimental design, biological replication, control setting, detection methods, and data completeness.Clinical risk-of-bias tools were not applicable.
**Effect measures**	12	Specify for each outcome the effect measure(s) (e.g. risk ratio, mean difference) used in the synthesis or presentation of results.	Page 4 Methods, metabolite fold change and gene relative expression values were collected; no quantitative meta-analysis was conducted.
**Synthesis methods**	13a	Describe the processes used to decide which studies were eligible for each synthesis (e.g. tabulating the study intervention characteristics and comparing against the planned groups for each synthesis (item 5)).	Page 4 Methods, included studies were grouped by light quality and plant taxonomic family for qualitative narrative synthesis.
	13b	Describe any methods required to prepare the data for presentation or synthesis, such as handling of missing summary statistics, or data conversions.	Page 4 Methods, incomplete missing experimental data were described textually without statistical conversion processing.

Topic	No.	Item	Location where item is reported
	13c	Describe any methods used to tabulate or visually display results of individual studies and syntheses.	Page 4 Methods. Results of individual studies were summarized in structured tables according to plant species, light quality, treatment conditions,target metabolites, and gene expression indicators.
	13d	Describe any methods used to synthesize results and provide a rationale for the choice(s). If meta-analysis was performed, describe the model(s), method(s) to identify the presence and extent of statistical heterogeneity, and software package(s) used.	Page 4 Methods, only qualitative narrative synthesis was adopted; no quantitative meta-analysis performed.
	13e	Describe any methods used to explore possible causes of heterogeneity among study results (e.g. subgroup analysis, meta-regression).	Page 4 Methods, studies grouped by light quality and plant family for qualitative inter-group comparison.
	13f	Describe any sensitivity analyses conducted to assess robustness of the synthesized results.	Page 4 Methods, no quantitative sensitivity analysis was conducted in this review.
**Reporting bias assessment**	14	Describe any methods used to assess risk of bias due to missing results in a synthesis (arising from reporting biases).	Both positive and negative light-response findings were considered where reported, in order to reduce selective interpretation of the evidence.
**Certainty assessment**	15	Describe any methods used to assess certainty (or confidence) in the body of evidence for an outcome.	Page 25 Discussion, evidence certainty judged by whether multi-omics combined functional verification was conducted.
**RESULTS**			

Topic	No.	Item	Location where item is reported
**Study selection**	16a	Describe the results of the search and selection process, from the number of records identified in the search to the number of studies included in the review, ideally using a flow diagram.	Page 4 Results & Figure 1 PRISMA flow diagram, full screening quantity data of all stages are listed.
	16b	Cite studies that might appear to meet the inclusion criteria, but which were excluded, and explain why they were excluded.	Page 4 & Supplementary Table S1, all excluded full-text papers with detailed exclusion reasons are listed.
**Study characteristics**	17	Cite each included study and present its characteristics.	Page 10—18 Table 1—3, basic information of all 50 included medicinal plant studies is summarized.
**Risk of bias in studies**	18	Present assessments of risk of bias for each included study.	Page 10—18 Table 1—3, experimental repeatability and data reliability of each study are narratively assessed.
**Results of individual studies**	19	For all outcomes, present, for each study: (a) summary statistics for each group (where appropriate) and (b) an effect estimate and its precision (e.g. confidence/credible interval), ideally using structured tables or plots.	Page 10—18 Table 1—3, metabolite accumulation and gene expression data of individual experiments are tabulated.
**Results of syntheses**	20a	For each synthesis, briefly summarise the characteristics and risk of bias among contributing studies.	Page 19—22 Results, studies grouped by light quality to summarize research features and experimental quality.

Topic	No.	Item	Location where item is reported
	20b	Present results of all statistical syntheses conducted. If meta-analysis was done, present for each the summary estimate and its precision (e.g. confidence/credible interval) and measures of statistical heterogeneity. If comparing groups, describe the direction of the effect.	Page 4 Methods, this review adopts qualitative narrative synthesis without quantitative meta-analysis.
	20c	Present results of all investigations of possible causes of heterogeneity among study results.	Page 19—22 Results, inter-group comparison across different light spectra explains heterogeneous metabolic responses.
	20d	Present results of all sensitivity analyses conducted to assess the robustness of the synthesized results.	Page 4 Methods, no quantitative sensitivity analysis was carried out in this review.
**Reporting biases**	21	Present assessments of risk of bias due to missing results (arising from reporting biases) for each synthesis assessed.	Page 24 Discussion, all positive and negative light-induced metabolite data were fully collected to minimize reporting bias.
**Certainty of evidence**	22	Present assessments of certainty (or confidence) in the body of evidence for each outcome assessed.	Page 25 Discussion, evidence certainty was graded by whether multi-omics combined functional verification was performed.
**DISCUSSION**			
**Discussion**	23a	Provide a general interpretation of the results in the context of other evidence.	Page 22—26 Discussion, our findings were cross-referenced and interpreted with published related systematic reviews.

Topic	No.	Item	Location where item is reported
	23b	Discuss any limitations of the evidence included in the review.	Page 25 Discussion, limitations of static single-spectrum light experimental evidence in existing studies were summarized.
	23c	Discuss any limitations of the review processes used.	Page 25 Discussion, the limitation of unavailable prospective PROSPERO registration for this review process was explained.
	23d	Discuss implications of the results for practice, policy, and future research.	Page 26 Future Perspectives, optimized dynamic light recipes and synthetic biology research directions for medicinal plants were proposed.
**OTHER INFORMATION**			
**Registration and protocol**	24a	Provide registration information for the review, including register name and registration number, or state that the review was not registered.	Page 3 Methods. This review was notprospectively registered in PROSPERO, OSF, or anyother systematic review registry.
	24b	Indicate where the review protocol can be accessed, or state that a protocol was not prepared.	No publicly accessible review protocol was prepared or registered before the conduct of this review.
	24c	Describe and explain any amendments to information provided at registration or in the protocol.	Not applicable, as no review protocol was prospectively registered.
**Support**	25	Describe sources of financial or non-financial support for the review, and the role of the funders or sponsors in the review.	Last page Funding section, all national and provincial supporting projects are listed; funders took no part in study design and manuscript writing.

Topic	No.	Item	Location where item is reported
**Competing interests**	26	Declare any competing interests of review authors.	Last page Conflicts of Interest section, all authors declare no competing financial or commercial interests.
**Availability of data, code and other materials**	27	Report which of the following are publicly available and where they can be found: template data collection forms; data extracted from included studies; data used for all analyses; analytic code; any other materials used in the review.	The PRISMA checklist and full search strategies are provided as supplementary files.No original analytic code was generated because this review used qualitative narrative synthesis rather than quantitative meta–analysis. Additional data are available from the corresponding author upon reasonable request.

PRIMSA Abstract Checklist

Topic	No.	Item	Reported?
**TITLE**			
**Title**	1	Identify the report as a systematic review.	Yes
**BACKGROUND**			
**Objectives**	2	Provide an explicit statement of the main objective(s) or question(s) the review addresses.	Yes
**METHODS**			
**Eligibility criteria**	3	Specify the inclusion and exclusion criteria for the review.	Yes
**Information sources**	4	Specify the information sources (e.g. databases, registers) used to identify studies and the date when each was last searched.	Yes
**Risk of bias**	5	Specify the methods used to assess risk of bias in the included studies.	No
**Synthesis of results**	6	Specify the methods used to present and synthesize results.	Yes
**RESULTS**			
**Included studies**	7	Give the total number of included studies and participants and summarise relevant characteristics of studies.	No
**Synthesis of results**	8	Present results for main outcomes, preferably indicating the number of included studies and participants for each. If meta-analysis was done, report the summary estimate and confidence/credible interval. If comparing groups, indicate the direction of the effect (i.e. which group is favoured).	Yes
**DISCUSSION**			

Topic	No.	Item	Reported?
**Limitations of evidence**	9	Provide a brief summary of the limitations of the evidence included in the review (e.g. study risk of bias, inconsistency and imprecision).	Yes
**Interpretation**	10	Provide a general interpretation of the results and important implications.	Yes
**OTHER**			
**Funding**	11	Specify the primary source of funding for the review.	Yes
**Registration**	12	Provide the register name and registration number.	No

From: Page MJ, McKenzie JE, Bossuyt PM, Boutron I, Hoffmann TC, Mulrow CD, et al. The PRISMA 2020 statement: an updated guideline for reporting systematic reviews. MetaArXiv. 2020, September 14. DOI: 10.31222/osf.io/v7gm2. For more information, visit: www.prisma-statement.org
